# TaADF3, an Actin-Depolymerizing Factor, Negatively Modulates Wheat Resistance Against *Puccinia striiformis*

**DOI:** 10.3389/fpls.2015.01214

**Published:** 2016-01-18

**Authors:** Chunlei Tang, Lin Deng, Dan Chang, Shuntao Chen, Xiaojie Wang, Zhensheng Kang

**Affiliations:** State Key Laboratory of Crop Stress Biology for Arid Areas and College of Plant Protection, Northwest A&F UniversityYangling, China

**Keywords:** actin depolymerizing factors, wheat, *Puccinia striiformis* f. sp. *tritici*, abiotic stress, ROS, fungal penetration, actin filaments

## Abstract

The actin cytoskeleton has been implicated in plant defense against pathogenic fungi, oomycetes, and bacteria. Actin depolymerizing factors (ADFs) are stimulus responsive actin cytoskeleton modulators. However, there is limited evidence linking ADFs with plant defense against pathogens. In this study, we have isolated and functionally characterized a stress-responsive ADF gene (*TaADF3*) from wheat, which was detectable in all examined wheat tissues. *TaADF3* is a three-copy gene located on chromosomes 5AL, 5BL, and 5DL. A particle bombardment assay in onion epidermal cells revealed the cytoplasmic and nuclear localization of TaADF3. The expression of *TaADF3* was inducible by abscisic acid (ABA), as well as various abiotic stresses (drought and cold) and virulent *Puccinia striiformis* f. sp. *tritici* (*Pst*) but was down regulated in response to avirulent *Pst*. Virus-induced silencing of *TaADF3* copies enhanced wheat resistance to avirulent *Pst*, with decreased reactive oxygen species (ROS) accumulation and hypersensitive response (HR). Upon treatment with virulent *Pst, TaADF3*-knockdown plants exhibited reduced susceptibility, which was accompanied by increased ROS production and HR. Interestingly, the silencing of *TaADF3* resulted in hindered pathogen penetration and haustoria formation for both avirulent and virulent *Pst*. Moreover, the array and distribution of actin filaments was transformed in *TaADF3*-knockdown epidermal cells, which possibly facilitated attenuating the fungus penetration. Thus, our findings suggest that *TaADF3* positively regulates wheat tolerance to abiotic stresses and negatively regulates wheat resistance to *Pst* in an ROS-dependent manner, possibly underlying the mechanism of impeding fungal penetration dependent on the actin architecture dynamics.

## Introduction

Actin is one of the most abundant and highly conserved proteins in eukaryotic cells. The dynamic reorganization and rearrangement of the actin cytoskeleton is associated with various important cellular processes that are essential for cell growth, differentiation, division, membrane organization, motility, cold acclimation, and wound repair (Pollard et al., 2000; Wasteneys and Galway, 2003; Day et al., 2011). Increasing evidence has shown that the actin cytoskeleton is precisely regulated to function as a contributing factor to plant immunity against pathogen ingress (Hardham et al., 2007; Tian et al., 2009; Henty-Ridilla et al., 2013). Pharmacological perturbation of the cytoskeleton compromised the basal defense and non-host resistance of a range of plants species by increasing the incidence of pathogen entry (Kobayashi et al., 1997a; Yun et al., 2003; Shimada et al., 2006; Miklis et al., 2007). The actin cytoskeleton also plays a role in race-specific resistance (Skalamera and Heath, 1998; Tian et al., 2009). The actin-based cytoskeleton is modulated by a plethora of actin-binding proteins (ABPs), among which the actin-depolymerizing factors (ADFs) and the cofilins form a single family called the ADF/cofilins (Bamburg, 1999). They are abundant and essential in almost every eukaryotic cell type and are responsible for the high turnover rates of actin filaments *in vivo* (Staiger et al., 1997; Dos Remedios et al., 2003; Van Troys et al., 2008). The interaction between actin and ADF/cofilins is controlled by reversible phosphorylation, ubiquitination, pH, oxidation, phosphoinositides, and specific proteins (Ayscough, 1998).

Whereas most non-plant organisms contain only one or two genes encoding ADF proteins, plant species appear to express larger families of ADF genes (Meagher et al., 1999). In terms of phylogenetic relationships, plant ADF/cofilins are classified into at least four groups (Mun et al., 2000). Group I is composed exclusively of dicots except for a rice ADF gene, whereas Group IV is proposed to be exclusive to the monocots (Danyluk et al., 1996). Group II and Group III are expressed in both dicots and monocots, although Group II is pollen specific (Lopez et al., 1996). Higher-plant ADFs exhibit specific temporal and spatial expression patterns, and the preferential tissue existence seems to be related to their distinct roles in different biological processes. Pollen-specific ADFs in Group II serve to bind and remodel F-actin in pollen grains in cooperation with other actin binding proteins (Lopez et al., 1996; Allwood et al., 2002; Chen et al., 2003). ADFs in root hairs function to increase the turnover of actin filaments (Jiang et al., 1997; Dong et al., 2001). In Arabidopsis, 12 ADFs in four ancient subclasses exhibit distinct tissue-specific and developmental expression and have been proposed to have different functions (Ruzicka et al., 2007). The diverse expression patterns and functions of ADFs appear to co-evolve with the ancient and divergent actin isovariants.

Corresponding to the regulatory role of the actin cytoskeleton in plants against various environmental stimuli, plant ADFs have been shown to play an important role in response to biological invasion and abiotic stress. ADFs from Arabidopsis, barley and wheat were found to be related to plant resistance to various pathogens (Miklis et al., 2007; Tian et al., 2009; Fu et al., 2014). The ectopic expression of barley *HvADF3* effectively impedes actin cytoskeleton integrity, thereby enhancing the susceptibility of the *Mlo* genotype to barley powdery mildew and partially breaks down *mlo* resistance with an elevated incidence of fungal entry (Miklis et al., 2007). The Arabidopsis AtADF4 is potentially targeted by the bacterial effector protein AvrPphB under the control of the cognate resistance gene RPS5-mediated resistance to *Pseudomonas syringae* (Porter et al., 2012). *AtADF4* mediated both effector-triggered immunity (ETI) and PAMP-triggered immunity (PTI) signaling due to its activity in actin rearrangement modulation or translocation of the cytoskeleton into the nucleus through the nuclear localization signal (NLS), where these triggers function as gene expression regulators (Tian et al., 2009; Porter et al., 2012; Henty-Ridilla et al., 2014). In wheat, *TaADF7* contributes to resistance against *Puccinia striiformis* f. sp. *tritici* (*Pst*) by modulating the cytoskeleton dynamics to influence ROS accumulation and HR (Fu et al., 2014). During cold acclimation, another wheat ADF protein, TaADF, accumulated to higher levels in freeze-tolerant but not sensitive wheat cultivars (Ouellet et al., 2001). Ectopic overexpression of *OsADF3* conferred enhanced drought/osmotic stress tolerance on transgenic Arabidopsis by modulating several downstream abiotic stress-responsive target genes related to drought responses (Huang et al., 2012).

As one of the top 10 plant-pathogenic fungi, *Pst* causes destructive wheat stripe rust disease worldwide (Dean et al., 2012). In response to *Pst* infection, wheat shows race-specific resistance accompanied with hypersensitive response (HR), rapid cell death at neighboring mesophyll cells and infected sites. As *Pst* is an obligate biotrophic basidiomycete, which could not be cultured *in vitro*, the wheat-*Pst* interaction mechanism has been largely hindered. The expanded understanding of the profound regulation of ADF/cofilins and the multifaceted functions of these ADF/cofilins in physiological changes has led to the conclusion that ADF/cofilin proteins are a functional node in cell biology (Bernstein and Bamburg, 2010). Despite their multiple and essential roles, there is still limited evidence linking ADFs with host pathogen defense, especially in the wheat-*Pst* interaction phytosystem, except for *TaADF7* (Fu et al., 2014). Similar to the presence of 12 ADF genes in the entire rice and Arabidopsis genomes, the wheat genome also encodes a large ADF family consisting of multiple ADF genes. In this study, we isolated a novel ADF gene, *TaADF3*, that encodes a protein sharing only 57.55% similarity to TaADF7. To investigate the function of *TaADF3* in wheat, we analyzed its spatial and temporal expression patterns under various exogenous stresses. Furthermore, knockdown of *TaADF3* in wheat was performed to analyze whether and how *TaADF3* participates in wheat resistance to *Pst*. Our results demonstrated that *TaADF3* positively regulates wheat tolerance to drought and cold, possibly by participating in the abscisic acid (ABA) signaling pathway. Further silencing analyses revealed that *TaADF3* negatively regulated wheat resistance to *Pst*, most likely by hindering fungus entry in an reactive oxygen species (ROS)-dependent manner. These findings provide new insight into the role of ADFs in host immunity to biotrophic fungal pathogens.

## Materials and methods

### Plant and fungal material

Wheat (*Triticum aestivum* L.) genotype Suwon 11 and *Pst* pathotypes CYR23 and CYR31 were used for this study. Wheat cv. Suwon 11 contains the stripe rust resistance gene *YrSu* (Cao et al., 2002) and is resistant to CYR23 but highly susceptible to CYR31. Wheat seedlings were grown, inoculated and maintained as described by Kang and Li (1984). *Pst* pathotypes CYR23 and CYR31 were maintained on wheat cv. Mingxian 169 and Suwon 11, respectively. The fresh uredinospores of CYR23 and CYR31 were inoculated on the first leaves of wheat cv. Suwon 11 at the first leaf stage. Parallel mock control plants were inoculated with sterile water. After inoculation, plants were kept in a dark chamber with 100% humidity for 24 h and subsequently transferred to a growth chamber at 15°C with a 16 h photoperiod under fluorescent white light. Wheat leaves were sampled at 0, 12, 18, 24, 48, 72, and 120 h post-inoculation (hpi).

For chemical treatment, 2-week-old wheat seedlings were sprayed with 2 mM salicylic acid (SA), 100 mM methyl jasmonate (MeJA), 100 mM ethepon, and 100 mM abscisic acid (ABA) dissolved in 0.1% (v/v) ethanol. Mock control plants were treated with 0.1% ethanol. The first leaves that were treated with chemicals along with the control plants were sampled at 0, 0.5, 2, 6, 12, and 24 h post-treatment (hpt). For various abiotic stresses, the roots of wheat seedlings were soaked in 200 mM NaCl or 20% PEG6000 for high salinity or drought treatment. To cause wounding, the first wheat leaves of 2-week-old seedlings were scraped with a sterilized needle. Low-temperature treatment was performed by transferring the wheat seedlings to a 4°C chamber. The first leaves of the treated plants and mock control plants were collected at 0, 2, 6, 12, 24, and 48 hpt. Intact tissues of different wheat organs from 2-week-old seedlings were collected for tissue-specific expression analysis, except for glume, which was collected at the adult stage of wheat seedlings.

All the freshly collected samples were immediately frozen into liquid nitrogen and stored at −80°C prior to the extraction of total RNA or DNA. For each time point, three independent biological replications were performed.

### RNA/DNA isolation and qRT-PCR

Genomic DNA of wheat leaves was extracted using the DNeasy Plant Mini Kit (Qiagen). Total RNA from wheat leaves treated with chemicals, challenged by abiotic stresses and *Pst*, and different wheat tissues were extracted using the RNeasy Plant Mini Kit (Qiagen) and treated with DNase I to remove the contaminating DNA. First strand cDNA was synthesized from 2 μg of total RNA using the SuperScript First-strand Synthesis System (Invitrogen, Carlsbad, CA, USA). The expression of the *TaADF3* gene was controlled using the wheat elongation factor *TaEF-1*α gene (GenBank accession no. Q03033). Quantitative RT-PCR was performed on a 7500 Real-time PCR system (Applied Biosystems, Foster City, CA, USA), and the relative gene expression was quantified using the comparative 2^−ΔΔCT^ method (Livak and Schmittgen, 2001). All reactions were performed in triplicate. The primers used for qRT-PCR are listed in Table S1.

### Cloning of *TaADF3* and sequence analyses

Based on the EST sequence (TA54178_4565) in the TIGR Wheat Genome Database, a set of primers TaADF3-cDNA-F and TaADF3-cDNA-R were designed to amplify *TaADF3* from the cDNA of wheat leaves. The DNA sequence was obtained by genomic PCR using the total DNA of wheat leaves as the template. The physical characteristics of the deduced protein encoded by the obtained cDNA were computed using the Compute pI/MW Tool. Multiple sequence alignments and phylogenetic analysis were conducted using DNAMAN and MEGA (version 4.0) software, respectively. The phylogram was constructed using the neighbor-joining method, in which bootstrap support values were based on 1000 replicates.

### Plasmid construction

For subcellular localization in onion cells, the TaADF3 protein-encoding sequence was amplified and inserted into the *Hin*dIII and *Nco*I sites of the *pCaMV35S::GFP* vector to generate the *pCaMV35S::TaADF3-GFP* fusion vector.

The plasmids used for the silencing of *TaADF3* in the barley stripe mosaic virus (BSMV)—mediated virus-induced gene silencing (VIGS) experiment were constructed as described previously (Holzberg et al., 2002). A cDNA fragment derived from the coding sequence and the 3′ untranslated region (416–592) was inserted into the *Not*I and *Pac*I sites to replace the phytoene desaturase (PDS) gene fragment of γ:*PDS* and generate the recombinant γ:*TaADF3*. To guarantee the specificity of gene silencing, the cDNA sequence of *TaADF3* was aligned with the *T. aestivum* cv. Chinese Spring (CS) genome using the service provided by the International Wheat Genome Sequencing Consortium (http://wheat-urgi.versailles.inra.fr/Seq-Repository/BLAST). The fragments that showed the highest polymorphism within the gene family and the lowest sequence similarity to other genes were chosen for constructing γRNA-based derivative plasmids.

### Subcellular localization

The fusion *pCaMV35S::TaADF3-GFP* construct and the control plasmid *pCaMV35S::GFP* were transformed into onion epidermal cells by particle bombardment at a helium pressure of 1100 psi using the PDS-1000/He system (Bio-Rad, Hercules, CA, USA). The transformed onion epidermal cells were cultured on MS medium plates at 28°C for 18–24 h in a dark chamber. Fluorescent signals were observed using a Zeiss LSM 510 confocal laser microscope (Zeiss, Germany) with a 480-nm filter.

### BSMV-mediated silencing of *TaADF3* in wheat cv. Suwon 11

By *in vitro* transcription using a high-yield capped RNA transcription kit (mMESSAGE mMACHINE; Ambion), BSMV RNAs were prepared from linearized plasmids. For inoculation, the RNA transcripts were diluted four times, and 2.5 μL of each transcript, including the BSMV RNA α, β, and γ (γ-*TaPDS*, γ-*TaADF3*) transcripts, were mixed with 42.5 μL of FES buffer (Pogue et al., 1998). The mixture was inoculated into the second leaves of wheat seedlings at the two-leaf stage by gently rubbing the surface with a gloved finger (Scofield et al., 2005). BSMV: 00 and BSMV: *TaPDS* were used as controls for BSMV infection. Wheat seedlings inoculated with FES buffer were used as the mock controls. The virus-infected wheat seedlings were kept in a growth chamber at 25 ± 2°C under a 16 h photoperiod. Ten days post BSMV inoculation, the fourth leaves were further inoculated with fresh uredinospores of *Pst* pathotype CYR23 or CYR31, and the plants were subsequently maintained as described above. Three independent sets of plants were prepared for each assay. The disease phenotype of the fourth leaves was observed and photographed 14 days post-inoculation of *Pst*.

### Expression level of *TaADF3* and pathogenesis related genes in *TaADF3*-knockdown plants

The fourth leaves inoculated with BSMV:00 or BSMV:*TaADF3* were collected at 0, 24, 48, and 120 h post-inoculation (hpi) with CYR23 or CYR31, as well as the mock control plants. The relative expression of *TaADF3* was analyzed by qRT-PCR in each assay to assess the silencing efficiency compared to the control plants. The relative transcription levels of pathogenesis-related protein (PR) genes *TaPR1* (AAK60565), *TaPR2* (DQ090946), and *TaPR5* (FG618781) in the *TaADF3*-silenced leaves were confirmed by qRT-PCR.

### Histological observation of host defense and fungal growth in *TaADF3*-knockdown plants

The defense response and fungal growth in *TaADF3*-knockdown plants were observed microscopically. For histological observation, leaf segments (1.5 cm in length) were fixed and decolorized in ethanol/ acetic acid (1:1 v/v). The specimens were cleared in saturated chloral hydrate until leaf tissue became translucent. The autofluorescence of the attacked mesophyll cells was observed under a fluorescence microscope (excitation filter 485 nm, dichromic mirror 510 nm, barrier filter 520 nm) and measured using DP-BSW software to determine the necrotic cell area. The H_2_O_2_ that accumulated in the infection sites was stained using 3,3′-diaminobenzidine (DAB; Amresco, Solon, OH, USA; Wang et al., 2007), viewed under differential interference contrast optics and measured using DP-BSW software. The infection structure of stripe rust fungus was stained by wheat germ agglutinin (WGA) conjugated to Alexa 488 (Invitrogen, Carlsbad, CA, USA), as previously described (Ayliffe et al., 2011). Leaf segements were autoclaved in 1 M KOH and 0.05% Silwet L-77 (Hood and Shew, 1996). After washing in 50 mM Tris (pH 7.5) twice, leaf tissue was stained with WGA-alexa (20 μg/ml) for 15 min. Then the tissues were rinsed with 50 mM Tris (pH 7.5) and mounted in 50 mM Tris (pH 7.5) to be examined under blue light excitation. The hyphal length, haustoria, and infection area were observed and calculated using DP-BSW software. Only infection sites where substomatal vesicles had formed underneath stomata were considered to be successful penetration and were microscopically evaluated the infection hyphae, haustoria, and infection area. Penetration success was calculated as the number of infection sites that exhibited one or multiple haustoria in relation to the total number of infection sites. Standard deviations and Student's *t*-test were applied for statistical analysis.

### Fungal biomass analyses in *TaADF3*-knockdown plants

Absolute quantification of the wheat stripe rust fungus in infected wheat leaves was analyzed by qRT-PCR. First-strand cDNA was synthesized using 2 μg of total RNA from *Pst* CYR31 uredinospores or *Pst*-infected leaves pre-inoculated with BSMV:00 or BSMV:*TaADF3*. The cDNA of uredinospores of *Pst* CYR31 diluted in a gradient was used to generate the standard curve. The cDNA of the *Pst* infected leaves of BSMV:00- or BSMV:*TaADF3*-inoculated plants at 24, 48, and 120 hpi were adjusted to 300 ng/μL. For the quantification of wheat stripe rust fungus, the constitutively expressed wheat stripe rust elongation factor gene *Pst-EF* was used (Yin et al., 2011). The standard curve was used to perform the absolute quantification of *Pst* in planta.

### Actin filament staining

Actin microfilaments were stained as described previously (Kobayashi et al., 1997b) with slight modifications (Opalski et al., 2005). Ten days post-virus inoculation, the fourth leaves of the virus-infected plants were collected. The leaf segments (5 × 5 mm in size) were fixed in 3.7% formaldehyde in 25 mM piperazine-N,N′-bis (2-ethanesulfonic acid) buffer (PIPES, pH 6.8), with 2 mM EGTA, 2 mM MgCl_2_, and 0.05% Tween 20 (v/v) at room temperature for 1 h. After washing in 25 mM PIPES and 25 mM phosphate buffer (PBS, pH 6.8), leaf segments were treated with 0.5% Triton X-100 in 25 mM PBS (pH 6.8) at room temperature for 1 h. The specimens were washed with 25 mM PBS (pH 6.8), then with 25 mM PBS (pH 7.4) for three times. Then leaf segments were stained with Alexa-Fluor 488 phalloidin (0.66 μM in 25 mM PBS, pH 7.4). Vacuum infiltration was performed three times for 20 s at 27 mm Hg to promote uptake of the dye. Subsequently, samples were stored at room temperature for 3 h in darkness. Finally, leaves were rinsed with 25 mM PBS (pH 7.4), mounted in 25 mM PBS (pH 7.4) on glass slides and observed by fluorescence microscopy. Three biological replications were performed and approximately five leaf segments were observed for each repliation.

### Statistical analyses

Mean values and standard errors were calculated with Microsoft Excel software. Statistical significance was assessed by one-tailed Student's *t*-test with unequal variance and between control and treatment.

## Results

### TaADF3 encodes an actin depolymerizing factor

Based on the EST sequence (TA54178_4565) in the wheat TIGR genome database, a cDNA fragment of 795 bp in length was obtained with an open reading frame (ORF) of 417 bp, which shows the highest similarity (97.83%) to the actin-depolymerizing factor 3 (GenBank no. AIZ95472.1) of *T. aestivum*. PCR amplification using the same primers obtained a genomic sequence of 1858 bp, consisting of three exons split by two introns with lengths of 972 bp and 91 bp. The first exon exclusively encodes the first start codon (methionine), typically found in ADF genes (Figure S1). BlastN analyses in the *T. aestivum* cv. Chinese spring (CS) genome sequence showed that there are three copies of this gene in the wheat genome, located on the long arms of chromosomes 5A, 5B, and 5D (Figure S1). The ADF gene obtained in this study and wheat actin-depolymerizing factor 3 in the NCBI Database exhibited the highest identity with the copies on chromosome 5BL and 5AL, respectively. The results indicate that these two genes are actually two homologous genes located on different chromosomes. Thus, here, we designated the ADF gene as *TaADF3*. The two copies of *TaADF3* on 5AL and 5DL encode the same protein, showing one residue variation from 5BL (Figure S2), although there were variations at 18 nucleotide positions in the open reading frames of the three copies (Figure S3).

**Figure 1:**
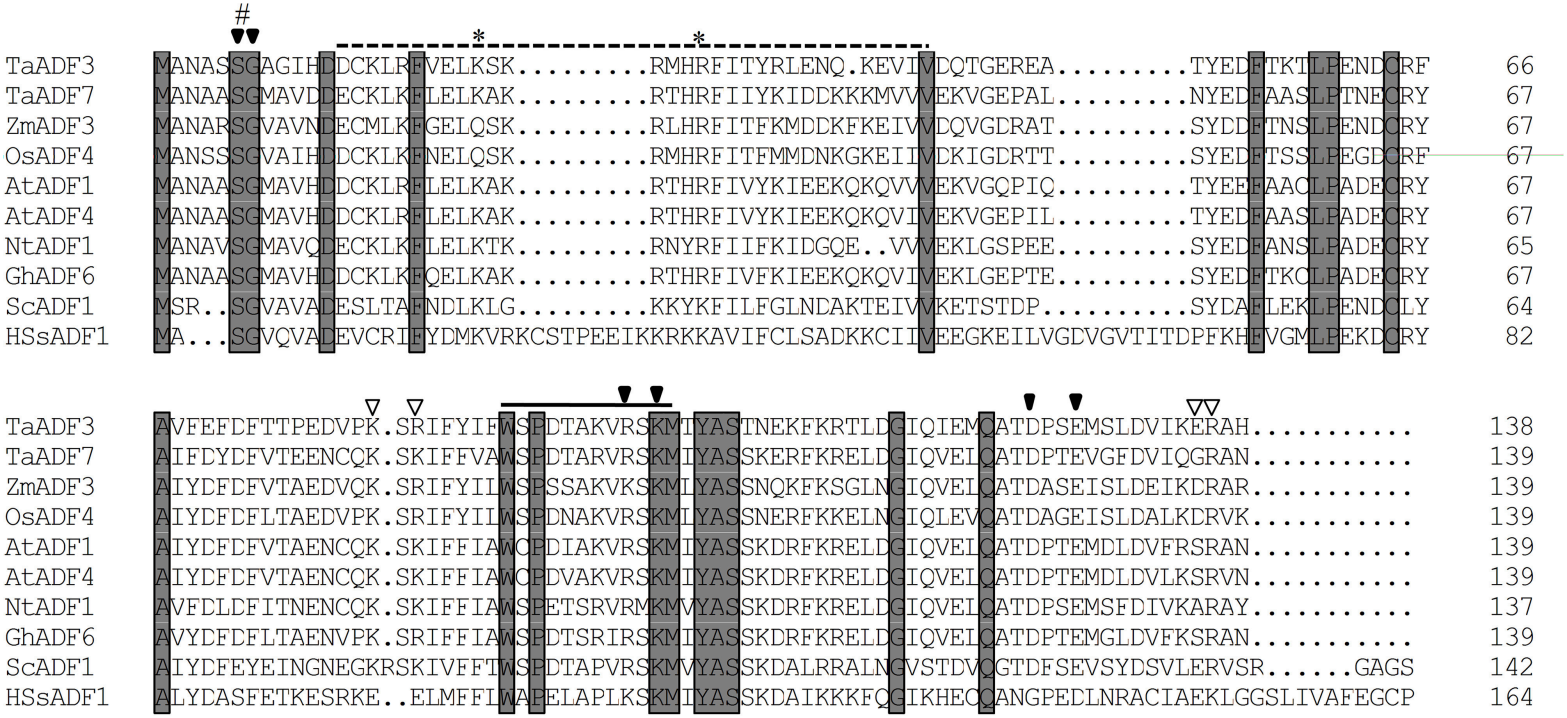
**Multiple alignment of TaADF3 against ADF/cofilins from other species**. Each ADF/cofilin contains preserved phosphorylation sites on serine (#) and a nucleus localization signal (^*^). The solid arrow and open arrow indicate the binding site of G-actin and F-actin, respectively. The dotted line represents the CAM combining region and the solid line indicates the PIP2/actin-binding domain. Ta, *Triticum aestivum*; Os, *Oryza sativa*; Zm, *Zea mays*; At, *Arabidopsis thaliana*; Nt, *Nicotiana tomentosiformis*; Gh, *Gossypium hirsutum*; Sc, *Saccharomyces cerevisiae*; Hs, *Homo sapiens*.

The deduced TaADF3 protein encoded 138 amino acid residues with a molecular weight of 16.10 kDa and an isoelectric point (PI) of 5.65. Multi-alignment of TaADF3 and ADFs from other higher plants revealed a preserved Ser6 in plant ADFs (Ser3 in animal) that could be phosphorylated. TaADF3 contained a bipartite NLS—Lys22 and Arg28 close to the amino terminus; Ser6, Gly7, Arg97, Lys99, Asp124, and Glu127 could bind to actin monomers (G-actin); Lys81, Arg83, Glu135, and Arg136 could specifically bind to microfilaments (F-actin). A CAM combining region (Asp13—Val42) and a PIP2 (phospholipid phosphatidylinositol -4, 5-bisphosphate) binding domain (Trp89—Met100) were also included in the sequence (Figure 1). The results indicate the conservation of ADF proteins across different higher plant species.

Based on the spatial and temporal expression pattern, the ADF proteins in higher plants were categorized into four groups. As shown in Figure S4, the majority of Group I ADF members are from dicotyledon plants, except for the ADF7 proteins from wheat, barley and *Brachypodium distachyon*. Group II contains pollen-specific ADFs, which can then be sub-grouped into the monocot group and the dicot group. Group III includes ADFs from both monocotyledons and dicotyledons. In contrast to the other groups, Group IV, to date, exclusively contains monocot ADFs and is most closely related to animal ADF/cofilins (Figure S4). Phylogenetic analyses showed that TaADF3 was homologous to rice OsADF4 and corn ZmADF3, with 67.63 and 62.59% similarity, respectively. In the phylogram, all of them belong to Group IV of the ADF/cofilin family.

### TaADF3 is localized in both cytoplasm and nucleus

**Figure 2:**
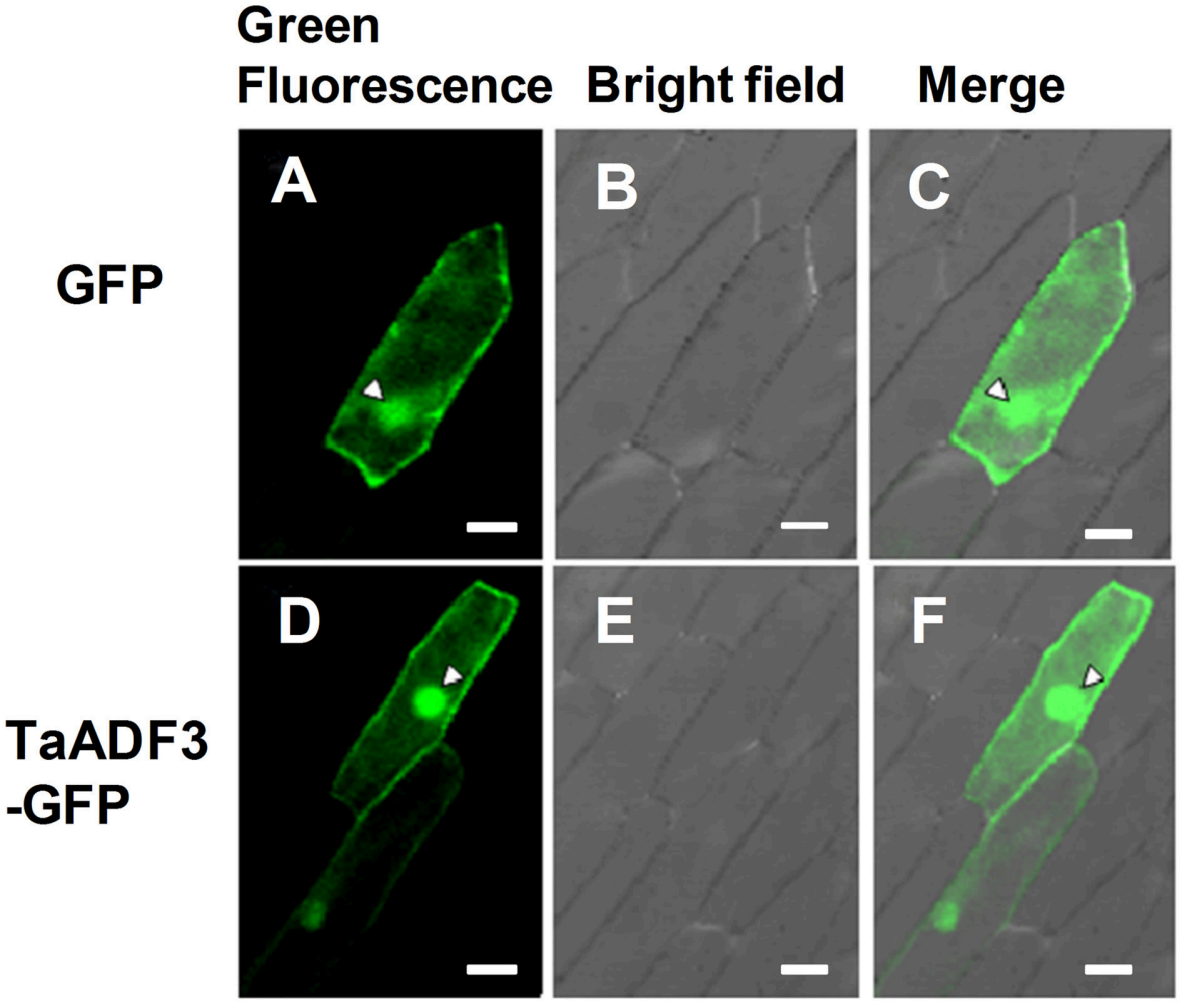
**Subcellular localization of TaADF3 in onion cells**. Green fluorescent protein (GFP) **(A–C)** or TaADF3-GFP fusion protein **(D–F)** was transiently expressed in onion epidermal cells under the control of the cauliflower mosaic virus (CaMV) 35S promoter by particle bombardment. The green fluorescence was observed under a confocal microscope and imaged using a 488-laser excitation light source **(A,D)**. The corresponding cell morphology photos were taken under bright field **(B,E)**. Bar, 50 μm.

To determine the subcellular localization of TaADF3, the fusion construct pCaMV35S::TaADF3-GFP was transiently expressed in onion epidermal cells by particle bombardment. Laser-scanning confocal micrographs showed the green fluorescence of fusion TaADF3-GFP protein in both cytoplasm and nucleus, the same as the distribution of GFP alone (Figure 2).

### Tissue-specific expression of *TaADF3*

**Figure 3:**
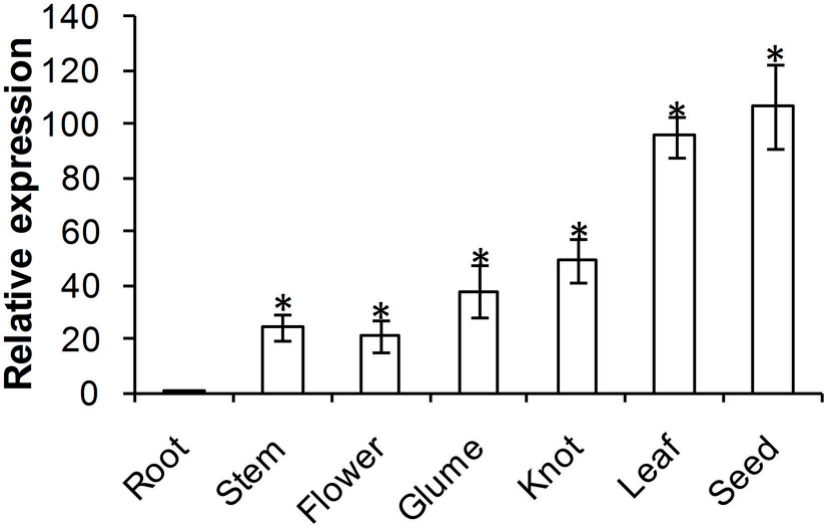
**Expression patterns of *TaADF3* in different wheat tissues**. Samples were collected from root, stem, flower, glume, knot, leaf, and seed. The relative expression level was analyzed by qRT-PCR and normalized to the wheat elongation factor *TaEF-1*α gene. Three independent biological replications were performed. Asterisks indicate a significant difference (*P* < 0.05) from the root using Student's *t*-test.

ADF protein in higher plants is reported to show tissue specific expression patterns. To examine the physiological role of *TaADF3*, the transcript of *TaADF3* in different wheat tissues was examined by qRT-PCR. The result showed that *TaADF3* was detectable in all tested wheat tissues, with the lowest level in the root. The *TaADF3* transcript is most abundant in wheat leaf and the developing seed by ~95 and 106 times the amount in the root. *TaADF3* transcript is also highly abundant in wheat stem, flower, glume and knot, although less than in leaf and seed (Figure 3).

### *TaADF3* is upregulated in response to abiotic stresses

**Figure 4:**
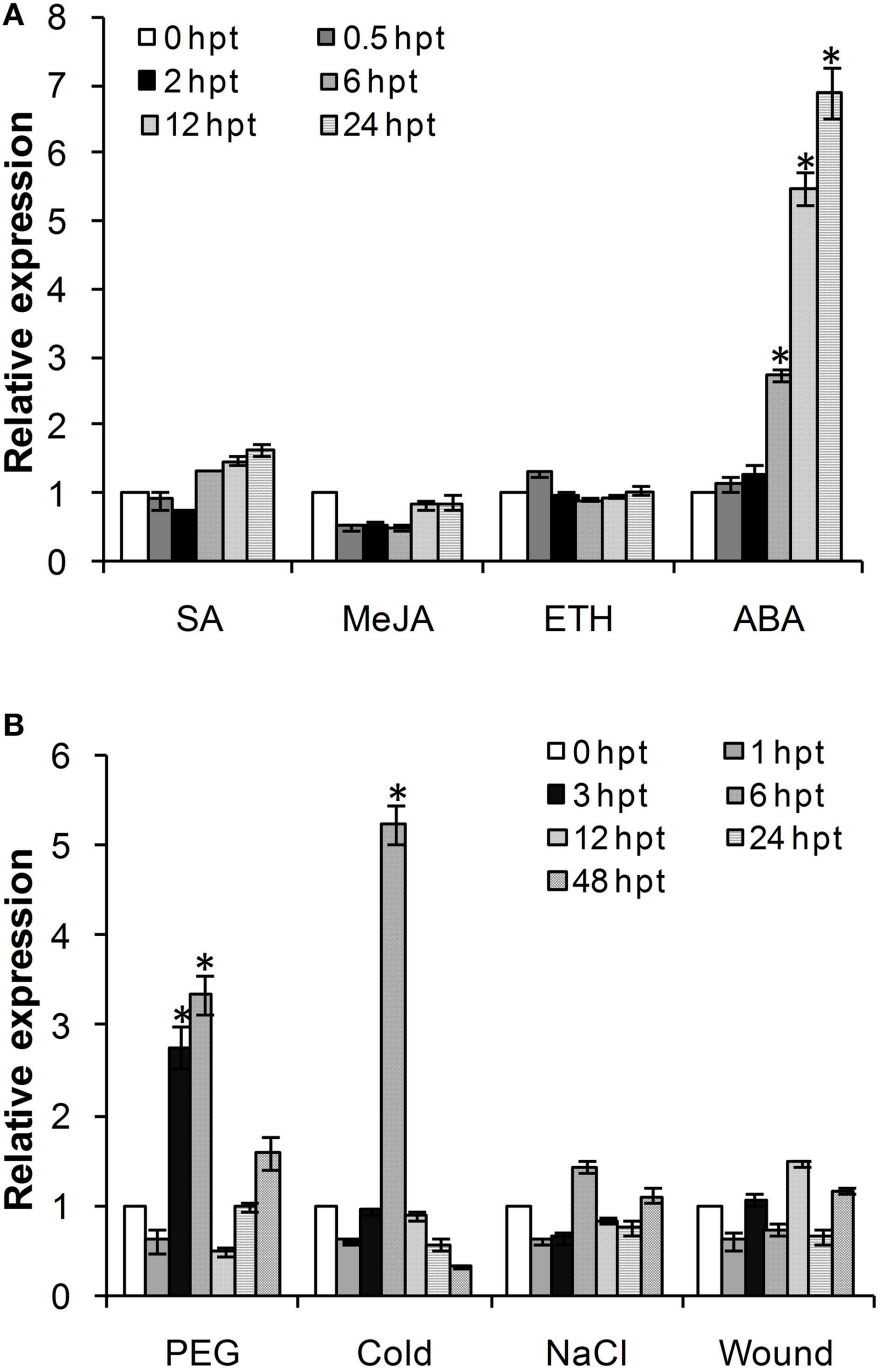
**Expression profile of *TaADF3* in response to exogenous hormones (A) and abiotic stresses (B)**. Three independent biological replications were performed. The expression levels were normalized to *TaEF-1*α, and the results are shown as the mean ± standard deviation of three biological replications. Asterisks indicate a significant difference (*P* < 0.05) from 0 hpt using Student's *t*-test. SA, salicylic acid; MeJA, methyl jasmonate; ETH, ethylene; ABA, abscisic acid.

Considering the involvement of ADFs in the response to various abiotic stresses, we investigated the effects of exogenous hormone chemicals and abiotic stresses on the expression of *TaADF3*. As shown in Figure 4A, *TaADF3* was mainly induced by ABA treatment but showed no significant response to the other treatments. In ABA treatment, the expression of *TaADF3* was continuously increased after 6 hpt (hour post-treatment) and peaked at 24 hpt with approximate 7-fold expression. These results suggested that *TaADF3* may be related to the ABA-dependent signaling pathway.

The transcriptional levels of *TaADF3* were also induced by some abiotic elicitors (Figure 4B). PEG6000 treatment and low temperature (4°C) could significantly upregulate the expression of *TaADF3*. Both treatments reached the peak at 6 hpt with approximately 3-fold and 5-fold increases, respectively. Compared with cold treatment, *TaADF3* was induced earlier under PEG6000 treatment. In contrast, under wounding and high salinity treatments, the expression of *TaADF3* did not exhibit any significant changes.

### *TaADF3* is induced upon virulent *Pst* attack

**Figure 5:**
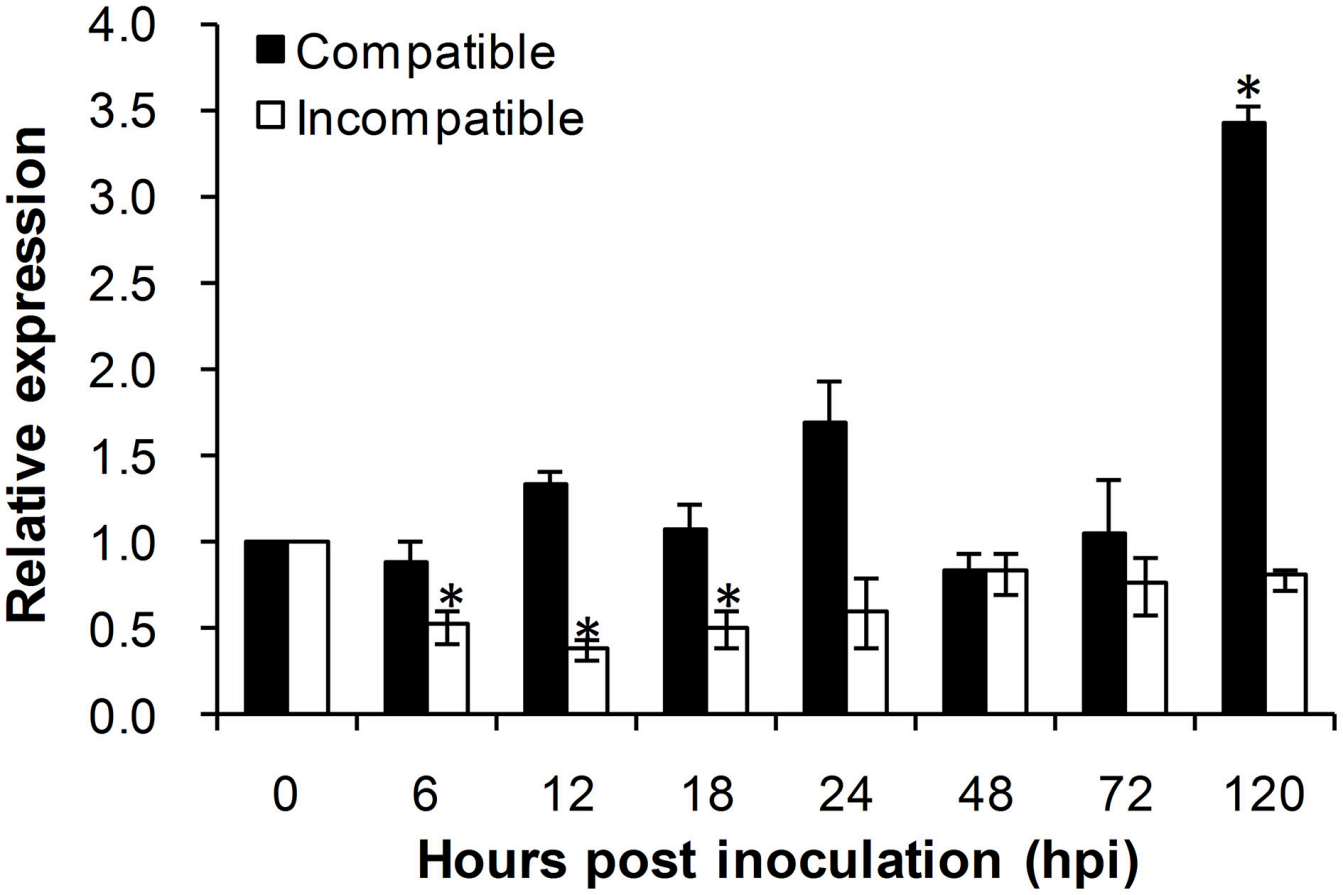
**Transcript profile of *TaADF3* in wheat leaves inoculated with virulent and avirulent *Pst* races**. In compatible interaction, wheat cultivar Suwon 11 was inoculated with virulent *Pst* CYR31, and in incompatible interaction, wheat Suwon 11 was challenged by avirulent *Pst* CYR23. The data were normalized to wheat *TaEF-1*α gene, and the results were obtained from three independent replicates. Vertical bars represent the standard deviation. Asterisks indicate a significant difference (*P* < 0.05) from 0 hpt using Student's *t*-test.

To investigate the role of *TaADF3* in plant-pathogen interactions, the transcriptional profile of *TaADF3* was determined in Suwon 11 wheat leaves inoculated with *Pst* pathotypes CYR31 and CYR23 for compatible and incompatible interactions, respectively. During wheat-*Pst* interaction, the transcript level of *TaADF3* was induced at 120 hpi in wheat leaves challenged by the virulent *Pst* pathotype CYR31, reaching a level 2.4-fold higher than that in the control plants (Figure 5). In wheat leaves challenged by the avirulent *Pst* pathotype CYR23, *TaADF3* was repressed as soon as the plants were infected by CYR23 (6 hpi) and had the lowest expression level (~0.4-fold) at 12 hpi. The significant difference between compatible and incompatible interactions (particularly at 12, 18, and 120 hpi) suggested that *TaADF3* may be a negative regulator in wheat defense against stripe rust fungus.

### Silencing of *TaADF3* enhances wheat resistance to *Pst*

**Figure 6:**
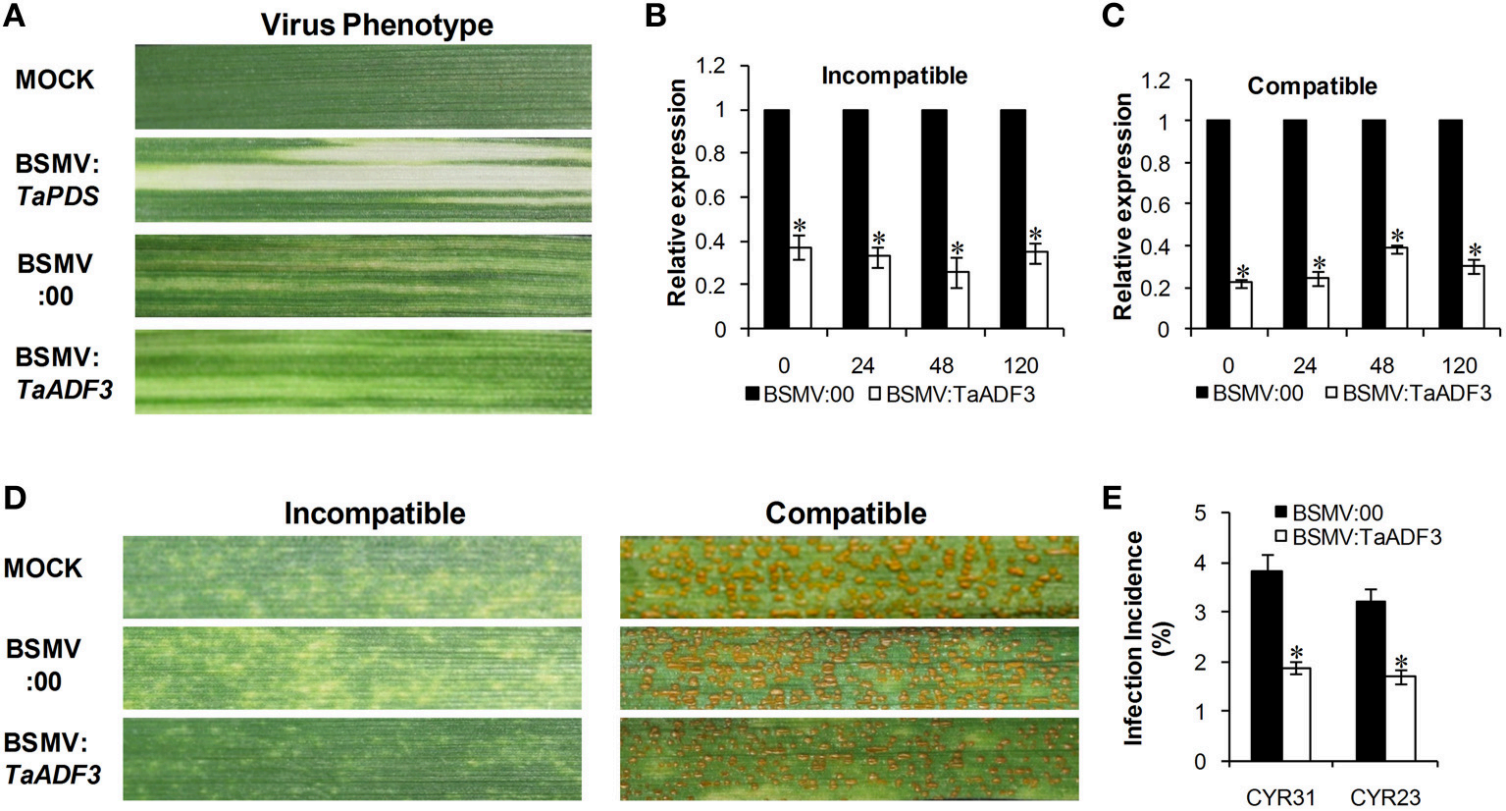
**Functional characterization of *TaADF3* during interaction of wheat and *Pst* by BSMV-mediated gene silencing. (A)** Photobleaching was evident on the fourth leaves of wheat plants inoculated with BSMV:*TaPDS*. Mild chlorotic virus symptoms were observed on the fourth leaves of wheat seedlings inoculated with BSMV:00 or BSMV:*TaADF3*. MOCK: wheat leaves inoculated with FES buffer. Silencing efficiency of *TaADF3* in the fourth leaves of *TaADF3*-knockdown plants in incompatible **(B)** or compatible **(C)** interaction. Wheat leaves inoculated with BSMV:00 and further challenged by stripe rust fungus were used as the controls. The data were normalized to the *TaEF-1*α gene. **(D)** Disease phenotypes of the fourth leaves further challenged by avirulent CYR23 or virulent CYR31. Photos were taken 14 days post pathogen inoculation. **(E)** Silencing of *TaADF3* attenuated infection of the virulent *Pst* CYR31 and the avirulent *Pst* CYR23. Only infection sites where substomatal vesicle formed were considered as successful penetration. The number of successful infection sites per 100 stoma was calculated. Three independent biological replications were performed, and 20 sets of infection incidence were measured for each biological replication. Asterisks indicate a significant difference (*P* < 0.05) from BSMV:00 using Student's *t*-test.

To further characterize the function of *TaADF3* in the wheat defense response to stripe rust fungus, BSMV-mediated VIGS was used to silence the expression of *TaADF3*. Ten days after BSMV inoculation, mild chlorotic mosaic symptoms appeared on the fourth leaves of infected wheat seedlings, and the BSMV:*TaPDS* inoculated plants exhibited strong photo-bleaching (Figure 6A). Fourteen days post pathogen infection, the disease phenotype was observed.

Silencing efficiency assessment by qRT-PCR showed that the expression level of *TaADF3* was greatly reduced to different extents in *TaADF3*-knockdown plants compared with the control plants, with an approximate reduction as high as 80% (Figures 6B,C). The fragment used for silencing is shown in Figure S1. Due to the high identity among the three copies, the three copies should be silenced simultaneously.

With the significantly repressed *TaADF3* expression, less necrosis was observed on wheat leaves from *TaADF3*-knockdown plants inoculated with *Pst* race CYR23, in contrast to the high necrosis observed in control plants (Figure 6D). When challenged by *Pst* race CYR31, leaves from the wild-type plants and BSMV:00-infected plants exhibited a fully susceptible phenotype. Leaves of the *TaADF3*-knockdown plants also exhibited a susceptible phenotype, but obvious necrotic cell death was observed, accompanied by reduced sporulation (Figure 6D).

The incidence of sites with substomatal vesicle formation underneath stoma was assessed in *TaADF3*-knockdown plants at 24 hpi. As shown in Figure 6E, in compatible interaction, the successful infection incidence of *Pst* CYR31 in *TaADF3*-knockdown plants was 1.89%, which was significantly lower than that in the control plants (3.83%). In incompatible interaction, the infection incidence of *Pst* CYR23 was also reduced by 1.51% compared to the controls (Figure 6E).

### Elevated defense response in *TaADF3*-knockdown plants

**Figure 7:**
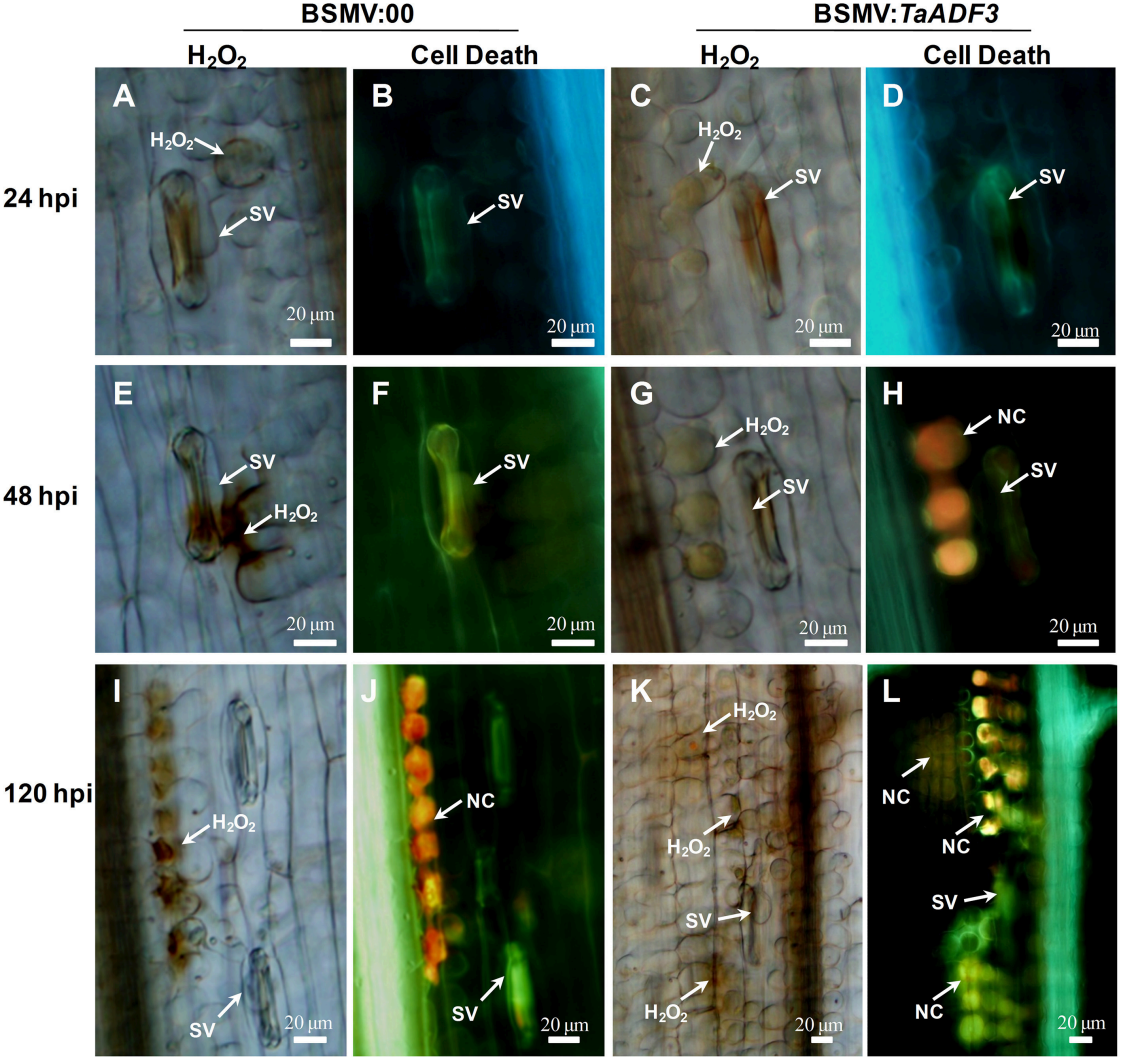
**Histological observation of the defense response in *TaADF3*-knockdown plants against the virulent *Pst* CYR31**. Wheat leaves that were pre-infected with BSMV:00 or recombinant BSMV:*TaADF3* were followed by *Pst* CYR31 inoculation. H_2_O_2_ burst and necrosis were observed in wheat leaves inoculated with BSMV:00 or BSMV:*TaADF3* at 24 hpi **(A–D)**, 48 hpi **(E–H)**, and 120 hpi **(I–L)**. Histochemical H_2_O_2_ accumulation at infection sites was stained using 3,3′-diaminobenzidine (DAB) staining and viewed under differential interference contrast optics. The autofluorescence of the attacked mesophyll cells at the same infection site was observed under a fluorescence microscope (excitation filter 485 nm, dichromic mirror 510 nm, barrier filter 520 nm). SV, substomatal vesicle; NC, necrotic cell death.

**Figure 8:**
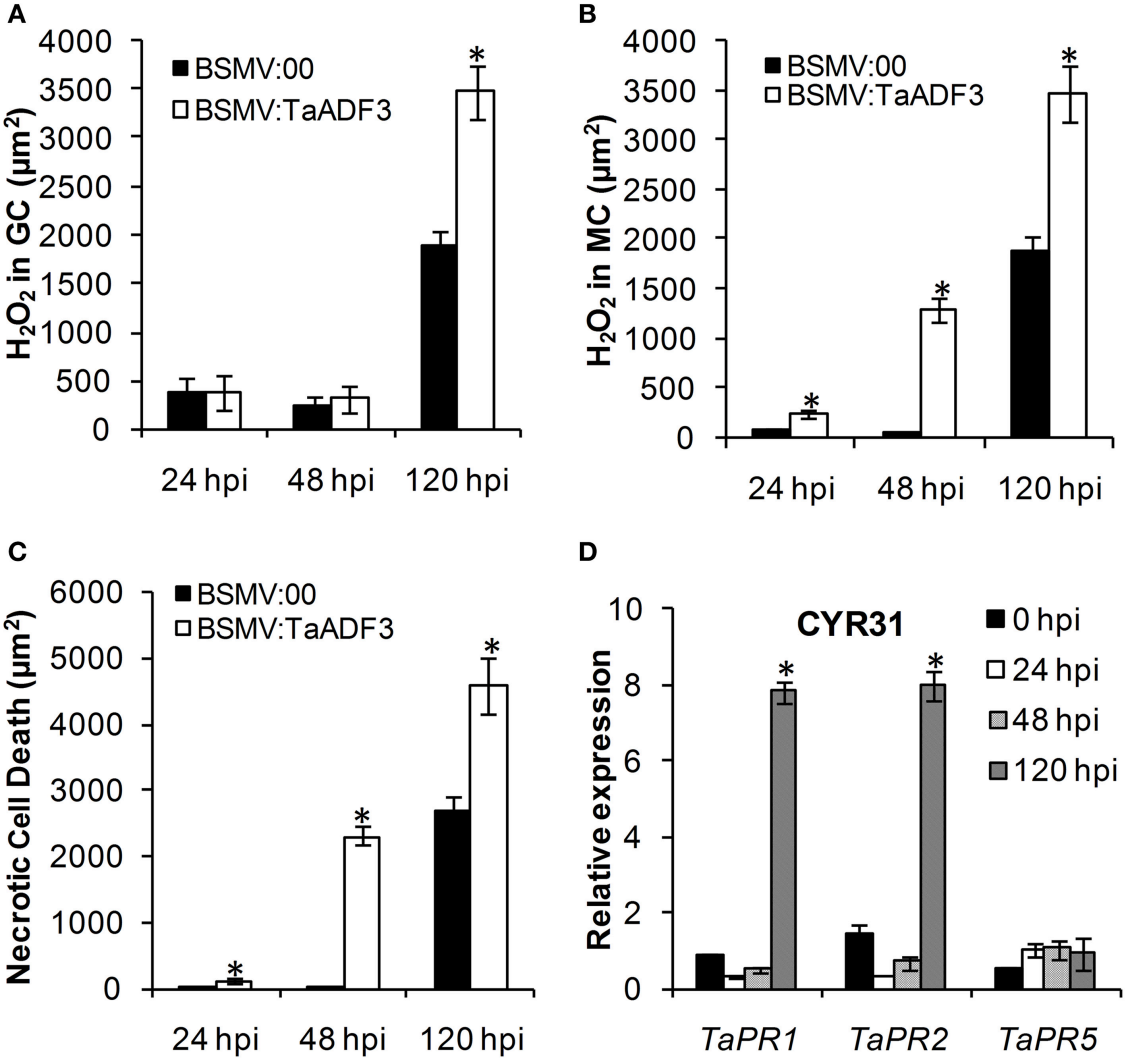
**Enhanced wheat defense response in *TaADF3*-knockdown plants attacked by virulent *Pst* CYR31**. The amount of H_2_O_2_ production was measured by calculating the DAB staining area at each infection site using the DP-BSW software **(A,B)**. The area of autofluorescence was measured to determine the necrotic cell death **(C)**. H_2_O_2_ produced in the guard cells (GC) and the attacked mesophyll cells (MC) was calculated. All results were obtained from 50 infection sites. **(D)** The expression profiles of three pathogenesis-related proteins were assessed in *TaADF3*-knockdown plants compared with the mock control plants. The data were normalized to the wheat *TaEF-1*α gene. Three independent biological replications were performed. Asterisks indicate a significant difference (*P* < 0.05) from BSMV:00 using Student's *t*-test.

Based on the observed enhanced resistance phenotype, the host response was further analyzed. We measured the H_2_O_2_ accumulation and necrotic cell death areas per infection site at 24, 48, and 120 hpi. In compatible interaction, H_2_O_2_ accumulation mainly occurred in the guard cells in the early stage, and the H_2_O_2_ amount in *TaADF3*-knockdown plants was not affected (Figures 7, 8A). At 24 and 48 hpi, H_2_O_2_ seldom occurred in mesophyll cells in mock control plants, which was also the case in *TaADF3*-knockdown plants at 24 hpi (Figure 7). However, at 48 hpi, 8.51% of the infection sites exhibited H_2_O_2_ production in attacked mesophyll cells when *TaADF3* was silenced, which was significantly higher than that in control plants (Figure S5A). Much more abundant H_2_O_2_ was accumulated in the attacked mesophyll cells (Figures 7, 8B). Along with the increased H_2_O_2_ at 48 hpi, the occurrence of necrosis and the corresponding necrosis area were significantly elevated (Figure 8C and Figure S5B). At 120 hpi, in control plants, obvious accumulation was already observed, but significantly increased H_2_O_2_ was accumulated in *TaADF3*-knockdown plants (Figures 7, 8C). The necrotic cell death observed by auto-fluorescence exhibited a similar increased pattern to the H_2_O_2_ accumulation (Figures 7, 8C).

In incompatible interaction, the H_2_O_2_ accumulation in guard cells was also not affected (Figure S6A), but the H_2_O_2_ accumulation in attacked mesophyll cells was decreased in *TaADF3*-knockdown plants, as was the necrosis area (Figures S6B,C). Lower occurrence of H_2_O_2_ production and cell death in mesophyll cells was observed in *TaADF3*-knockdown plants compared to the controls (Figures S5C,D). The reductions in observed cell death occurrence and area appear to correlate with the smaller observed necrosis.

Aside from the altered ROS accumulation and cell death, qRT-PCR analyses showed that *TaPR1* and *TaPR2* were sharply induced at 120 hpi in *TaADF3*-knockdwon plants attacked by either virulent CYR31 or avirulent CYR23 (Figure 8D and Figure S6D). In contrast to this dramatic induction, *TaPR5* was slightly induced only in the interaction with CYR23 (Figure 8D and Figure S6D). Taken together, all results suggested that the silencing of *TaADF3* enhanced the wheat defense response to *Pst*.

### Fungal entry and haustoria formation are impeded in *TaADF3*-knockdown plants

**Figure 9:**
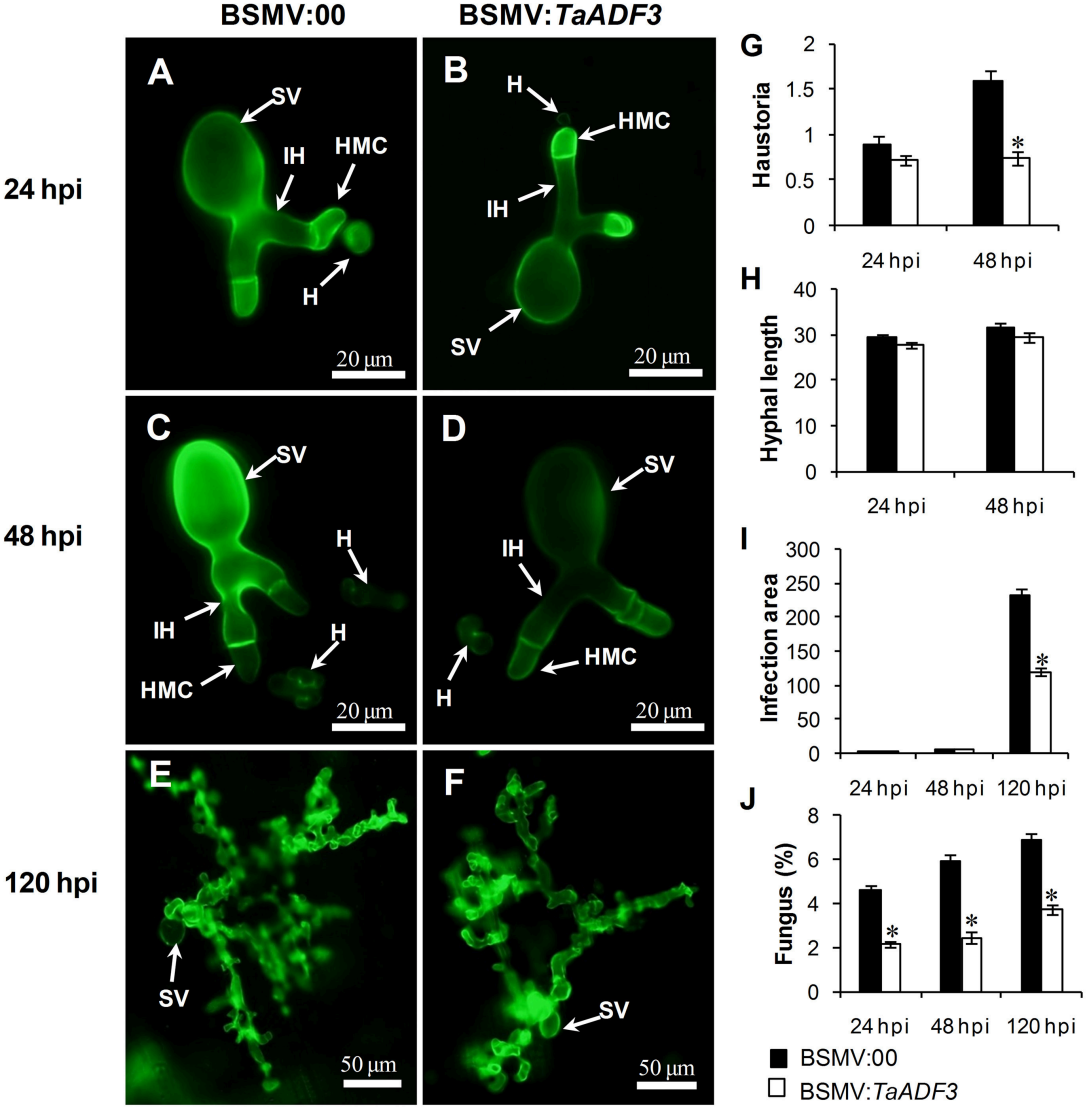
**Histological observation of fungal growth in *TaADF3*-knockdown plants challenged by virulent *Pst* CYR31**. The fungal structure was stained with wheat germ agglutinin (WGA). The fungal growth of *Pst* pathotype CYR31 in wheat leaves inoculated with BSMV:00 or BSMV:*TaADF3* at 24 hpi **(A,B)**, 48 hpi **(C,D)**, and 120 hpi **(E,F)** was observed under a fluorescence microscope. The average number of haustoria **(G)** of *Pst* in each infection site were counted. **(H)** Hyphal length, which is the average distance from the junction of the substomatal vesicle and the hypha to the tip of the hypha, was measured using DP-BSW software (unit in μm). **(I)** Infection area, the average area of the expanding hypha, was calculated using DP-BSW software (units of 10^3^ μm^2^). All results were obtained from 50 infection sites, and three biological replications were performed. **(J)** Quantification of fungus in *Pst* infected wheat leaves. The ratio of *Pst* CYR31 mRNA to total wheat mRNA was evaluated by qRT-PCR. Asterisks indicate a significant difference (*P* < 0.05) from BSMV:00-inoculated plants using a one-tailed Student's *t*-test. SV, substomatal vesicle; HMC, haustorial mother cell; IH, infection hypha; H, haustorium.

To test whether the enhanced resistance of wheat affected the survival of wheat stripe rust fungus, the growth and development of *Pst* were assessed through histological observation. As shown in Figure 9, in compatible interaction, the silencing of *TaADF3* greatly decreased the number of haustoria at 48 hpi (Figures 9A–D,G). Hyphal growth and infection area were not significantly affected at 24 and 48 hpi (Figures 9H,I). By 120 hpi, when haustoria formed in great numbers, the infected area of *Pst* was significantly smaller than in the controls (Figure 9I). Absolute quantification revealed less fungus in planta throughout the examined infection stages when *TaADF3* was silenced (Figure 9J).

In incompatible interaction, the knockdown of *TaADF3* caused decreased haustoria formation numbers at 48 hpi (Figure S7A), and the hyphal length and infection area were not affected at all (Figures S7B,C). The qRT-PCR analyses revealed a slight decrease in the fungal amount in infected *TaADF3*-knockdown plants (Figure S7D).

**Figure 10:**
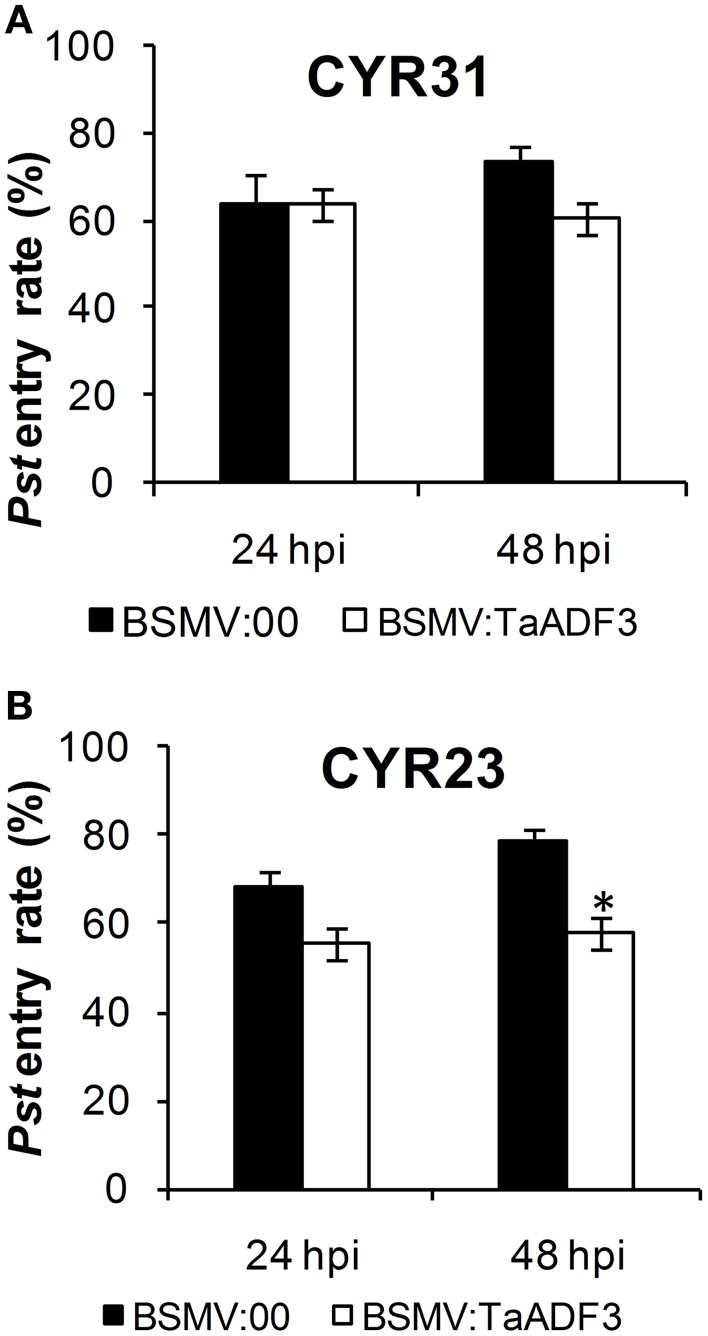
**Knockdown of the expression of *TaADF3* compromised fungus entry**. Successful fungus entry was assessed in *TaADF3*-knockdown plants, which were subsequently infected with virulent *Pst* CYR31 **(A)** or avirulent *Pst* CYR31 **(B)**. Only infection sites where substomatal vesicles had formed over stomata were considered successful penetration and were microscopically observed for the presence or absence of haustoria. Entry success was calculated as the number of penetration sites that exhibited one or multiple haustoria in relation to the total number of infection sites. The data shown represent the mean ±SD from at least three experiments in which, as a minimum, 50 successful penetration sites each were evaluated. Asterisks beside columns indicate *P* < 0.05 (Student's *t*-test) compared with the negative control (BSMV:00).

Furthermore, in compatible interaction, the fungal entry of *Pst* CYR31 in *TaADF3*-knockdown plants was reduced by 12.4% at 48 hpi and unchanged at 24 hpi (Figure 10A). In incompatible interaction, the successful entry of *Pst* CYR23 was reduced by 12.5 and 21.9% at 24 and 48 hpi, respectively (Figure 10B).

### Actin architecture rearrangement in *TaADF3*-knockdown plants

**Figure 11:**
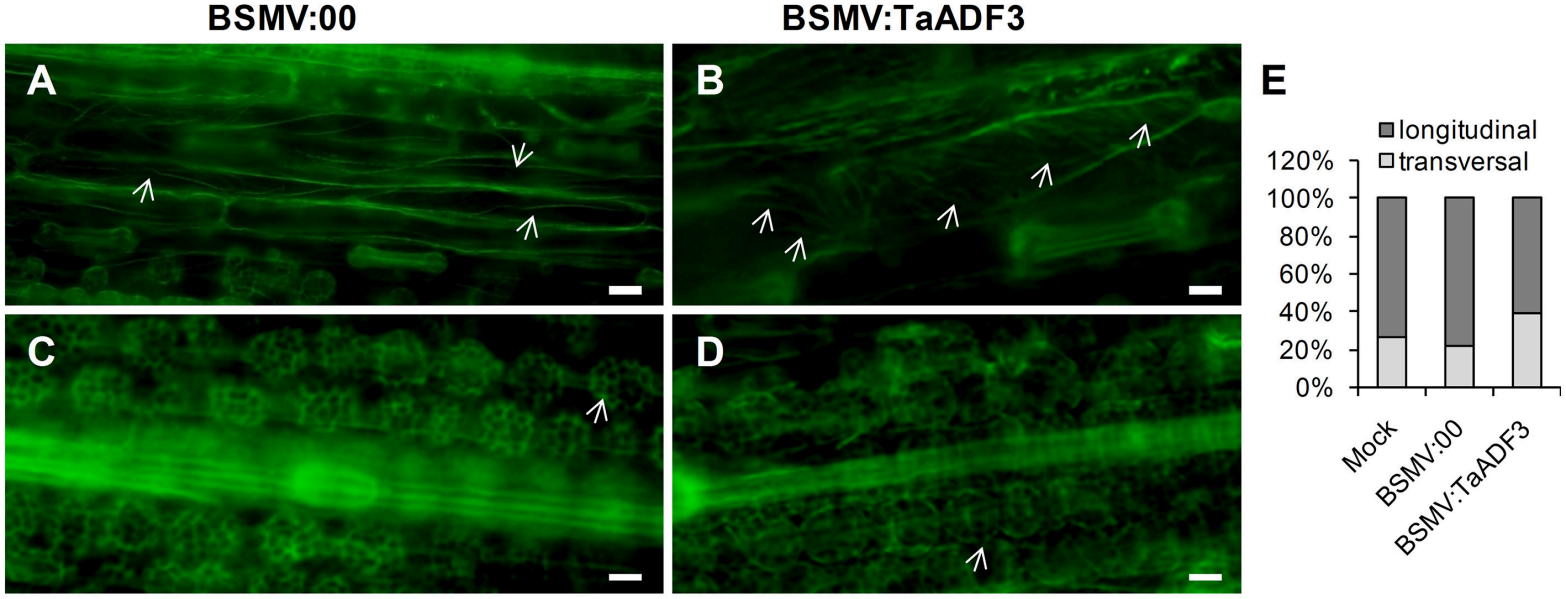
**Actin filament patterns in *TaADF3*-knockdown wheat cells**. Ten days post-virus inoculation, the fourth leaves of BSMV:00 or BSMV:TaADF3 inoculated plants were collected and stained with Alexa-Fluor 488 phalloidin, and observed under fluorescence microscopy. The stained F-actin was observed in epidermal cells **(A,B)** and mesophyll cells **(C,D)**. In epidermal cells, the actin filaments were arranged almost longitudinally or obliquely to the longitudinal axes of the cells **(A)**, or formed parallel arrays arranged obliquely or transversely to the longitudinal axis of the cells **(B)**. In mesophyll cells, the actin architecture in *TaADF3*-knockdown plants **(D)** did not seem to change significantly compared to the control cells **(C)**. **(E)** The proportion of actin filaments arranged in longitudinal array and transversal array was calculated. The data were obtained from at least 100 epidermal cells of five leaf segments and three biological replications were performed. The arrows indicate the Alexa-Fluor -labeled F-actin. Bar = 20 μm.

To examine whether silencing of *TaADF3* affects the actin cytoskeleton in wheat cells, 10 days post-virus inoculation, the fourth leaves were sampled for Alexa-Fluor 488 phalloidin staining. Alexa-Fluor-stained actin filaments were observed in wheat epidermal cells (Figures 11A,B) and mesophyll cells (Figures 11C,D). In epidermal cells, the actin filaments were observed in two different array patterns, as thin filamentous structures arranged almost longitudinally or obliquely to the longitudinal axes of the cells (Figure 11A), or formed parallel arrays arranged obliquely or transversely to the longitudinal axis of the cells (Figure 11B). In BSMV:00 infected cells, the actin filamentous in 78.4% of the observed epidermal cells was arranged in the longitudinal array, only 21.6% in transversal array (Figure 11E). In contrast, the transversely arranged actin filaments were observed in ~39.8% of the *TaADF3*-knockdown cells (Figure 11E), almost one-fold higher than that in control cells. Besides, the actin filaments appear to be more abundant in transversal array. In mesophyll cells, staining of actin filaments revealed an intact actin filament network in control cells (Figure 11C). Similarly, integrate caged actin architecture in *TaADF3*-knockdown plants was observed (Figure 11D).

## Discussion

ADFs are among the most highly expressed actin binding proteins that regulate actin dynamics. The four classified groups of ADFs in higher plants implies the functional divergence in the ADF family (Mun et al., 2002). The specific existence of Group IV ADF members in monocot plants may suggest their distinct roles (Maciver and Hussey, 2002). In this study, we isolated a wheat ADF gene, *TaADF3*, belonging to Group IV. The induced expression of *TaADF3* under PEG6000 treatment and low temperature indicated the involvement of *TaADF3* in enhancing plant acclimation to abiotic stresses. It has been reported that a wheat ADF member (TaADF) contributes to wheat cold acclimation regulated by genes located on chromosome 5A that are associated with cold hardiness (Ouellet et al., 2001). In addition, TaADF functions as a substrate for a wheat kinase, the activity of which is modulated by low temperature (Lopez et al., 1996). It is possible that *TaADF3* contributes to cold tolerance by interacting with other proteins to modulate cell cytoskeleton dynamics.

Plants perceive and respond adaptively to abiotic stress imposed by salt, cold, drought, and wounding, and the adaptive process is controlled mainly by the phytohormone ABA, which acts as an endogenous messenger in the regulation of plant water status (Swamy and Smith, 1999; Tuteja, 2007). The induction of *TaADF3* upon exogenous ABA application suggested that *TaADF3* may function as the downstream component in the ABA signaling pathway to elevate plant tolerance to abiotic stresses. ABA treatment is believed to result in stomatal closure through the disassembly of actin filaments (Eun and Lee, 1997). Thus, it can be assumed that *TaADF3* may participate in the ABA signaling pathway under abiotic stresses through regulating the actin dynamics in wheat stomatal movement. The ABA-dependent stomatal closure is also likely to function as a pre-invasive defense barrier against pathogens. Despite of the positive role of ABA in pre-invasive defense, its role in post-invasive defense seems to be mostly negative (Ton et al., 2009). Taking into account of the induced expression pattern of *TaADF3* under virulent *Pst*, it is reasonable that *TaADF3* is engaged in the negative regulation of post-invasive defense against *Pst* mediated by ABA, rather than contribute to the early pre-invasive defense.

ADFs have been implicated to play an important role in determining plant resistance against pathogenic microbes (Hardham et al., 2007). *HvADF3* was demonstrated to mediate race-specific immune responses in barley to an appropriate powdery mildew pathogen (Miklis et al., 2007). In the wheat-*Pst* interaction pathosystem, *TaADF7* was demonstrated to positively contribute to wheat resistance to *Pst* (Fu et al., 2014). In contrast to *TaADF7, TaADF3* function as a negative regulator in wheat resistance to *Pst*. Silencing of *TaADF3* enhanced race-specific immunity to *Pst* in *TaADF3*-knockdown plants. In response to the virulent *Pst* CYR31, *TaADF3*-knockdown plants was less susceptible with increased HR cell death, ROS accumulation and less sporulation. Previous histological and cytological observations revealed the oxidative bursts in the early (12–24 h) and late (96–120 h) infection stages of *Pst* (Wang et al., 2007). The induced *TaADF3* expression at 120 hpi in compatible interaction may be responsible for the suppression of ROS production. Thus, silencing of *TaADF3* led to increased ROS accumulation in *TaADF3*-kncokdown plants. It seemed that *TaADF3* negatively regulated wheat resistance in an ROS-dependent manner. Nevertheless, upon avirulent *Pst* infection, *TaADF3*-kncokdown plants retained complete resistance, but with less HR and ROS, which is closer to the immune response. The Arabidopsis *AtADF1-4* RNAi lines exhibited suppressed HR mediated by AvrPphB but retained the disease resistance phenotype (Tian et al., 2009). It appeared that HR can be uncoupled from resistance and that HR is not always for gene-for gene resistance. However, it is still possible that *TaADF3* may function in a dose-dependent manner to amplify defense signals. According to the hypothesis described by Jones and Dangl (2006), effective resistance or HR is achieved only when the amplitude of the defense signal reaches a certain threshold. We infer that the residual transcript of *TaADF3* was sufficient to sustain disease resistance but insufficient to attain the threshold for eliciting strong HR, as observed for *AtADF4* (Tian et al., 2009).

It has been documented that the actin cytoskeleton plays a crucial role in resistance during early stages of fungal penetration (Hardham et al., 2007; Miklis et al., 2007). The ectopic expression of *HvADF3* in barley leaf epidermal cells confers enhanced fungal entry of the powdery mildew fungus by interfering with the integrity of the plant actin cytoskeleton (Miklis et al., 2007). In our study, silencing of *TaADF3* resulted in transformed array of the actin filaments, which presumably serve as tracks for deposition of callose, secretion of antimicrobial products and delivery of vectorial vesicle (Henty-Ridilla et al., 2014). Based on the finding that *Pst* germinates on the leaf surface and penetrate through the stoma, it is tempting to speculate that the altered actin architecture partially attenuated *Pst* infection. The observed decreased haustoria number may be partially attributed to the impeded fungal entry. As the unique infection structure of biotrophic pathogens in host cells, the haustorium makes intimate contact with the host cell membrane and allows nutrient uptake and effector release into host cells (Voegele and Mendgen, 2003). The resulting compromised haustoria formation of *Pst* in *TaADF3*-knockdown plants would lead to a repressed nutrient supply, further limiting the growth and expansion of *Pst*. Whether the hindered *Pst* entry was due to the role of *TaADF3* on actin architecture is still unclear, although it seems that no detectable change was observed in mesophyll cells actin filaments.

In conclusion, this study demonstrated that *TaADF3* can positively modulate plant acclimation to abiotic stresses, possibly as a downstream component of ABA signaling pathway. Moreover, it suggested that *TaADF3* functions as a negative regulator in wheat resistance to *Pst* dependent on interfering the actin architecture, via limiting ROS release and hindering pathogen penetration. Nevertheless, the exact functional mechanism of *TaADF3* still needs further exploration.

## Author contributions

CT, LD, and ZK designed the experiment. CT, LD, DC and SC performed the experiments and analyzed the data. XW helped with data interpretation and article editing. CT wrote the manuscript.

### Conflict of interest statement

The authors declare that the research was conducted in the absence of any commercial or financial relationships that could be construed as a potential conflict of interest.

## Supplementary material

Table S1 **Oligonucleotides used for PCR and plasmid construction**.

Figure S1 **Gene structure of *TaADF3***. TaADF3, the full-length cDNA sequence amplified from cDNA of wheat Suwon 11; TaADF3-Genome, the genomic DNA sequence amplified from the total genomic DNA of wheat Suwon 11. The genome sequences of the three copies of *TaADF3* on chromosomes 5AL, 5BL, and 5DL were obtained from the wheat UGRI genome database of the wheat cultivar Chinese Spring. The full genome sequence of *TaADF3* contains three exons and two introns. The start codon and stop codon are indicated by red boxes, and the two introns are indicated by blue boxes. The specific fragment used for silencing is indicated by a red line.

Figure S2 **Multi-alignment of the deduced protein of the three copies of *TaADF3***.

Figure S3 **Multi-alignment of the encoding sequence of the three copies of *TaADF3***.

Figure S4 **Phylogenetic analyses of *TaADF3* and ADF members in other species**. Branches are labeled with protein names and GenBank accession numbers. Ta, *Triticum aestivum*; Bd, *Brachypodium distachyon*; Os, *Oryza sativa*; Zm, *Zea mays*; At, *Arabidopsis thaliana*; Nt, *Nicotiana tomentosiformis*; Gh, *Gossypium hirsutum*; Ph, *Petunia x hybrid*; Sc, *Saccharomyces cerevisiae*; Hs, *Homo sapiens*.

Figure S5 **Incidence of H_2_O_2_ production and cell death in *Pst* infected *TaADF3*-knockdown plants**. The fourth leaves of wheat seedlings inoculated with BSMV:00 or BSMV:*TaADF3* were further challenged by virulent or avirulent *Pst*. The H_2_O_2_ accumulation in attacked mesophyll cells in each infection site was observed under differential interference contrast optics through DAB staining. Necrotic cell death of the mesophyll cells at the same infection site was viewed under a flurorescence microscope. The number of infection sites with H_2_O_2_ accumulation and necrosis in attacked mesophyll cells among 50 infection sites was calculated. The incidence of H_2_O_2_ production and necrosis in mesophyll cells attacked by virulent Pst CYR31 **(A,B)** or avirulent Pst CYR23 **(C,D)** was measured. Three independent biological replications were performed. Asterisks indicate a significant difference (*P* < 0.05) from BSMV:00 using Student's *t*-test.

Figure S6 **Decreased H_2_O_2_ production and cell death in *TaADF3*-knockdown plant challenged by avirulent *Pst* CYR23**. The amount of H_2_O_2_ production was measured by calculating the DAB staining area at each infection site using DP-BSW software **(A,B)**. The area of autofluorescence was measured to determine the necrotic cell death **(C)**. H_2_O_2_ produced in guard cells (GC) and the attacked mesophyll cells (MC). All results were obtained from 50 infection sites. **(D)** The expression profiles of three pathogenesis-related proteins were assessed in *TaADF3*-knockdown plants compared withmock control plants. The data were normalized to the wheat *TaEF-1*α gene. Three independent biological replications were performed. Asterisks indicate a significant difference (*P* < 0.05) from BSMV:00 using Student's *t*-test.

Figure S7 **Fungal growth in *TaADF3*-knockdown plant challenged by avirulent *Pst* CYR23. (A)** Average numbers of haustoria of *Pst* CYR23 in each infection site were counted. **(B)** Hyphal length, which is the average distance from the junction of the substomatal vesicle and the hypha to the tip of the hypha, was measured using DP-BSW software (unit in μm). **(C)** Infection area, the average area of the expanding hypha, was calculated using DP-BSW software (units of 10^3^ μm^2^). All results were obtained from 50 infection sites, and three biological replications were performed. **(D)** Quantification of fungus in *Pst*-infected wheat leaves. The ratio of *Pst* CYR23 mRNA to total wheat mRNA was evaluated by qRT-PCR. Asterisks indicate a significant difference (*P* < 0.05) from BSMV:00-inoculated plants using a one-tailed Student's *t*-test.

## References

[B1] Allwood E. G. Anthony R. G. Smertenko A. P. Reichelt S. Drobak B. K. Doonan J. H.. (2002). Regulation of the pollen-specific actin-depolymerizing factor *LlADF1*. Plant Cell 14, 2915–2927. 10.1105/tpc.005363 12417710 PMC152736

[B2] Ayliffe M. Devilla R. Mago R. White R. Talbot M. Pryor A.. (2011). Nonhost resistance of rice to rust pathogens. Mol. Plant-Microbe Interact. 24, 1143–1155. 10.1094/MPMI-04-11-0100 21899436

[B3] Ayscough K. R. (1998). *In vivo* functions of actin-binding proteins. Curr. Opin. Cell Biol. 10, 102–111. 10.1016/S0955-0674(98)80092-6 9484601

[B4] Bamburg J. R. (1999). Proteins of the ADF/cofilin family: essential regulators of actin dynamics. Annu. Rev. Cell Dev. Biol. 15, 185–230. 10.1146/annurev.cellbio.15.1.185 10611961

[B5] Bernstein B. W. Bamburg J. R. (2010). ADF/cofilin: a functional node in cell biology. Trends Cell Biol. 20, 187–195. 10.1016/j.tcb.2010.01.001 20133134 PMC2849908

[B6] Cao Z. Jing J. Wang M. Shang H. Li Z. (2002). Relation analysis of stripe rust resistance gene in wheat important cultivar Suwon 11, Suwon 92 and hybrid 46. Acta Bot. BorealiOccident. Sin. 23, 64–68.

[B7] Chen Y.-H. Cheung A. Y. Wu H.-M. (2003). Actin-depolymerizing factor mediates Rac/Rop GTPase–regulated pollen tube growth. Plant Cell 15, 237–249. 10.1105/tpc.007153 12509534 PMC143494

[B8] Danyluk J. Carpentier E. Sarhan F. (1996). Identification and characterization of a low temperature regulated gene encoding an actin-binding protein from wheat. FEBS Lett. 389, 324–327. 10.1016/0014-5793(96)00599-6 8766725

[B9] Day B. Henty J. L. Porter K. J. Staiger C. J. (2011). The pathogen-actin connection: a platform for defense signaling in plants. Annu. Rev. Phytopathol. 49, 483–506. 10.1146/annurev-phyto-072910-095426 21495845

[B10] Dean R. Van Kan J. A. Pretorius Z. A. Hammond−Kosack K. E. Di Pietro A. Spanu P. D.. (2012). The top 10 fungal pathogens in molecular plant pathology. Mol. Plant Pathol. 13, 414–430. 10.1111/j.1364-3703.2011.00783.x 22471698 PMC6638784

[B11] Dong C. H. Xia G. X. Hong Y. Ramachandran S. Kost B. Chua N. H. (2001). ADF proteins are involved in the control of flowering and regulate F-actin organization, cell expansion, and organ growth in Arabidopsis. Plant Cell 13, 1333–1346. 10.1105/tpc.13.6.1333 11402164 PMC135580

[B12] Dos Remedios C. G. Chhabra D. Kekic M. Dedova I. V. Tsubakihara M. Berry D. A.. (2003). Actin binding proteins: regulation of cytoskeletal microfilaments. Physiol. Rev. 83, 433–473. 10.1152/physrev.00026.2002 12663865

[B13] Eun S. O. Lee Y. (1997). Actin filaments of guard cells are reorganized in response to light and abscisic acid. Plant Physiol. 115, 1491–1498. 10.1104/pp.115.4.1491 9414559 PMC158614

[B14] Fu Y. Duan X. Tang C. Li X. Voegele R. T. Wang X.. (2014). TaADF7, an actin-depolymerizing factor, contributes to wheat resistance against *Puccinia striiformis* f. sp. tritici. Plant J. 78, 16–30. 10.1111/tpj.12457 24635700

[B15] Hardham A. R. Jones D. A. Takemoto D. (2007). Cytoskeleton and cell wall function in penetration resistance. Curr. Opin. Plant Biol. 10, 342–348. 10.1016/j.pbi.2007.05.001 17627866

[B16] Henty-Ridilla J. L. Li J. Day B. Staiger C. J. (2014). ACTIN DEPOLYMERIZING FACTOR4 regulates actin dynamics during innate immune signaling in Arabidopsis. Plant Cell 26, 340–352. 10.1105/tpc.113.122499 24464292 PMC3963580

[B17] Henty-Ridilla J. L. Shimono M. Li J. Chang J. H. Day B. Staiger C. J. (2013). The plant actin cytoskeleton responds to signals from microbe-associated molecular patterns. PLoS Pathog. 9:e1003290. 10.1371/journal.ppat.1003290 23593000 PMC3616984

[B18] Holzberg S. Brosio P. Gross C. Pogue G. P. (2002). Barley stripe mosaic virus-induced gene silencing in a monocot plant. Plant J. 30, 315–327. 10.1046/j.1365-313X.2002.01291.x 12000679

[B19] Hood M. Shew H. (1996). Applications of KOH-aniline blue fluorescence in the study of plant-fungal interactions. Phytopathology 86, 704–708. 10.1094/Phyto-86-704

[B20] Huang Y. C. Huang W. L. Hong C. Y. Lur H. S. Chang M. C. (2012). Comprehensive analysis of differentially expressed rice actin depolymerizing factor gene family and heterologous overexpression of *OsADF3* confers *Arabidopsis thaliana* drought tolerance. Rice 5:33. 10.1186/1939-8433-5-33 24279948 PMC4883719

[B21] Jiang C. J. Weeds A. G. Hussey P. J. (1997). The maize actin-depolymerizing factor, ZmADF3, redistributes to the growing tip of elongating root hairs and can be induced to translocate into the nucleus with actin. Plant J. 12, 1035–1043. 10.1046/j.1365-313X.1997.12051035.x 9418045

[B22] Jones J. D. Dangl J. L. (2006). The plant immune system. Nature 444, 323–329. 10.1038/nature05286 17108957

[B23] Kang Z. Li Z. (1984). Discovery of a normal T. type new pathogenic strain to Lovrin10. Acta Cllegii Septentrionali Occident. Agric. 4, 18–28.

[B24] Kobayashi T. Shimanuki S. Saitoh S. Yamashita Y. (1997a). Improved growth of large lead zinc niobate titanate piezoelectric single crystals for medical ultrasonic transducers. Jpn. J. Appl. Phys. 36, 6035.

[B25] Kobayashi Y. Kobayashi I. Funaki Y. Fujimoto S. Takemoto T. Kunoh H. (1997b). Dynamic reorganization of microfilaments and microtubules is necessary for the expression of non-host resistance in barley coleoptile cells. Plant J. 11, 525–537.

[B26] Livak K. J. Schmittgen T. D. (2001). Analysis of relative gene expression data using real-time quantitative PCR and the 2^−Δ*ΔCT*^ method. Methods 25, 402–408. 10.1006/meth.2001.1262 11846609

[B27] Lopez I. Anthony R. G. MacIver S. K. Jiang C. J. Khan S. Weeds A. G.. (1996). Pollen specific expression of maize genes encoding actin depolymerizing factor-like proteins. Proc. Natl. Acad. Sci. U.S.A. 93, 7415–7420. 10.1073/pnas.93.14.7415 8693008 PMC38999

[B28] Maciver S. K. Hussey P. J. (2002). The ADF/cofilin family: actin-remodeling proteins. Genome Biol. 3, 3007.3001–3007.3012. 10.1186/gb-2002-3-5-reviews3007 12049672 PMC139363

[B29] Meagher R. B. McKinney E. C. Vitale A. V. (1999). The evolution of new structures: clues from plant cytoskeletal genes. Trends Genet. 15, 278–284. 10.1016/S0168-9525(99)01759-X 10390627

[B30] Miklis M. Consonni C. Bhat R. A. Lipka V. Schulze-Lefert P. Panstruga R. (2007). Barley MLO modulates actin-dependent and actin-independent antifungal defense pathways at the cell periphery. Plant Physiol. 144, 1132–1143. 10.1104/pp.107.098897 17449647 PMC1914182

[B31] Mun J. H. Lee S. Y. Yu H. J. Jeong Y. M. Shin M. Y. Kim H.. (2002). Petunia actin-depolymerizing factor is mainly accumulated in vascular tissue and its gene expression is enhanced by the first intron. Gene 292, 233–243. 10.1016/S0378-1119(02)00646-7 12119118

[B32] Mun J. H. Yu H. J. Lee H. S. Kwon Y. M. Lee J. S. Lee I.. (2000). Two closely related cDNAs encoding actin-depolymerizing factors of petunia are mainly expressed in vegetative tissues. Gene 257, 167–176. 10.1016/S0378-1119(00)00412-1 11080583

[B33] Opalski K. S. Schultheiss H. Kogel K. H. Hückelhoven R. (2005). The receptor-like MLO protein and the RAC/ROP family G-protein RACB modulate actin reorganization in barley attacked by the biotrophic powdery mildew fungus *Blumeria graminis* f. sp. hordei. Plant J. 41, 291–303. 10.1111/j.1365-313X.2004.02292.x 15634205

[B34] Ouellet F. Carpentier É. Cope M. J. T. Monroy A. F. Sarhan F. (2001). Regulation of a wheat actin-depolymerizing factor during cold acclimation. Plant Physiol. 125, 360–368. 10.1104/pp.125.1.360 11154343 PMC61016

[B35] Pogue G. P. Lindbo J. A. Dawson W. O. Turpen T. H. (1998). Tobamovirus transient expression vectors: tools for plant biology and high-level expression of foreign proteins in plants, in Plant Molecular Biology Manual, ed Gelvin S. B. (Dordrecht: Springer), 67–93.

[B36] Pollard T. D. Blanchoin L. Mullins R. D. (2000). Molecular mechanisms controlling actin filament dynamics in nonmuscle cells. Annu. Rev. Biophys. Biomol. Struct. 29, 545–576. 10.1146/annurev.biophys.29.1.545 10940259

[B37] Porter K. Shimono M. Tian M. Day B. (2012). Arabidopsis Actin-Depolymerizing Factor-4 links pathogen perception, defense activation and transcription to cytoskeletal dynamics. PLoS Pathog. 8:e1003006. 10.1371/journal.ppat.1003006 23144618 PMC3493479

[B38] Ruzicka D. R. Kandasamy M. K. McKinney E. C. Burgos-Rivera B. Meagher R. B. (2007). The ancient subclasses of Arabidopsis ACTIN DEPOLYMERIZING FACTOR genes exhibit novel and differential expression. Plant J. 52, 460–472. 10.1111/j.1365-313X.2007.03257.x 17877706

[B39] Scofield S. R. Huang L. Brandt A. S. Gill B. S. (2005). Development of a virus-induced gene-silencing system for hexaploid wheat and its use in functional analysis of the *Lr21*-mediated leaf rust resistance pathway. Plant Physiol. 138, 2165–2173. 10.1104/pp.105.061861 16024691 PMC1183404

[B40] Shimada C. Lipka V. O'Connell R. Okuno T. Schulze-Lefert P. Takano Y. (2006). Nonhost resistance in Arabidopsis-*Colletotrichum* interactions acts at the cell periphery and requires actin filament function. Mol. Plant-Microbe Interact. 19, 270–279. 10.1094/MPMI-19-0270 16570657

[B41] Skalamera D. Heath M. C. (1998). Changes in the cytoskeleton accompanying infection-induced nuclear movements and the hypersensitive response in plant cells invaded by rust fungi. Plant J. 16, 191–200. 10.1046/j.1365-313x.1998.00285.x 22507136

[B42] Staiger C. J. Gibbon B. C. Kovar D. R. Zonia L. E. (1997). Profilin and actin-depolymerizing factor: modulators of actin organization in plants. Trends Plant Sci. 2, 275–281. 10.1016/S1360-1385(97)86350-9

[B43] Swamy P. Smith B. (1999). Role of abscisic acid in plant stress tolerance. Curr. Sci. 76, 1220–1227.

[B44] Tian M. Chaudhry F. Ruzicka D. R. Meagher R. B. Staiger C. J. Day B. (2009). Arabidopsis actin-depolymerizing factor *AtADF4* mediates defense signal transduction triggered by the *Pseudomonas syringae* effector AvrPphB. Plant Physiol. 150, 815–824. 10.1104/pp.109.137604 19346440 PMC2689984

[B45] Ton J. Flors V. Mauch-Mani B. (2009). The multifaceted role of ABA in disease resistance. Trends Plant Sci. 14, 310–317. 10.1016/j.tplants.2009.03.006 19443266

[B46] Tuteja N. (2007). Abscisic acid and abiotic stress signaling. Plant Signal. Behav. 2, 135–138. 10.4161/psb.2.3.4156 19516981 PMC2634038

[B47] Van Troys M. Huyck L. Leyman S. Dhaese S. Vandekerkhove J. Ampe C. (2008). Ins and outs of ADF/cofilin activity and regulation. Eur. J. Cell Biol. 87, 649–667. 10.1016/j.ejcb.2008.04.001 18499298

[B48] Voegele R. T. Mendgen K. (2003). Rust haustoria: nutrient uptake and beyond. New Phytol. 159, 93–100. 10.1046/j.1469-8137.2003.00761.x 33873671

[B49] Wang C. F. Huang L. L. Buchenauer H. Han Q. M. Zhang H. C. Kang Z. S. (2007). Histochemical studies on the accumulation of reactive oxygen species (O^−2^ and H_2_O_2_) in the incompatible and compatible interaction of wheat: *Puccinia striiformis* f. sp. tritici. Physiol. Mol. Plant Pathol. 71, 230–239. 10.1016/j.pmpp.2008.02.006

[B50] Wasteneys G. O. Galway M. E. (2003). Remodeling the cytoskeleton for growth and form: an overview with some new views. Annu. Rev. Plant Biol. 54, 691–722. 10.1146/annurev.arplant.54.031902.134818 14503008

[B51] Yin C. Jurgenson J. E. Hulbert S. H. (2011). Development of a host-induced RNAi system in the wheat stripe rust fungus *Puccinia striiformis* f. sp. tritici. Mol. Plant-Microbe Interact. 24, 554–561. 10.1094/MPMI-10-10-0229 21190437

[B52] Yun B. W. Atkinson H. A. Gaborit C. Greenland A. Read N. D. Pallas J. A.. (2003). Loss of actin cytoskeletal function and EDS1 activity, in combination, severely compromises non-host resistance in Arabidopsis against wheat powdery mildew. Plant J. 34, 768–777. 10.1046/j.1365-313X.2003.01773.x 12795697

