# Supplementary material for: TaADF3, an Actin-Depolymerizing Factor, Negatively Modulates Wheat Resistance Against Puccinia striiformis
Source: Front Plant Sci. 2016 Jan 18;6:1214. doi: 10.3389/fpls.2015.01214 (PMC4716666; doi:10.3389/fpls.2015.01214)
Supplement: Table S1 — Oligonucleotides used for PCR and plasmid construction. [file Table1.docx]

**SupplementaryTable 1**. Oligonucleotides used for PCR and plasmid construction.

| **Applications** | **Primers** | **Sequence 5' to 3'** |
| --- | --- | --- |
| cDNA and DNA amplification | TaADF3-cDNA-F | AGCTACGCAGCACACAACC |
|  | TaADF3-cDNA-R | CTGACGAATCCAAACAGAACAT |
| qRT-PCR | TaADF3-F | ATAACCTACAGGCTGGAGAACC |
|  | TaADF3-R | TCCTGCTCTTTGGCACATC |
|  | TaPR1-F | GAGAATGCAGACGCCCAAGC |
|  | TaPR1-R | CTGGAGCTTGCAGTCGTTGATC |
|  | TaPR2-F | AGGATGTTGCTTCCATGTTTGCCG |
|  | TaPR2-R | AAGTAGATGCGCATGCCGTTGATG |
|  | TaPR5-F | CAAGCAGTGGTATCAACGCAGAG |
|  | TaPR5-R | GTGAAGCCACAGTTGTTCTTGATGTT |
|  | TaEF-1a-F | TGGTGTCATCAAGCCTGGTATGGT |
|  | TaEF-1a-F | ACTCATGGTGCATCTCAACGGACT |
| Fungus biomass | PstEF1-F | ATGCGTATCATGGTGGTGGAGTGA |
|  | PstEF1-R | TTCGCCGTCCGTGATATGAGACAA |
| Subcellular localization | TaADF3- *Hind*III-F | CCCAAGCTTATGGCAAACGCTTCATCAGGAG |
|  | TaADF3- *Nco*I-R | CATGCCATGGGTGCGCGCGCTCCTTGAT |
| VIGS | TaADF3-VIGS-F | ATATTAATTAACGAGCACCAACGAGAAGTTC |
|  | TaADF3-VIGS-R pET41a-AS | TATGCGGCCGCCAAGAGTCACACCGCCAAAT |
