# Supplementary figures and images for: TaADF3, an Actin-Depolymerizing Factor, Negatively Modulates Wheat Resistance Against Puccinia striiformis
Source: Front Plant Sci. 2016 Jan 18;6:1214. doi: 10.3389/fpls.2015.01214 (PMC4716666; doi:10.3389/fpls.2015.01214)

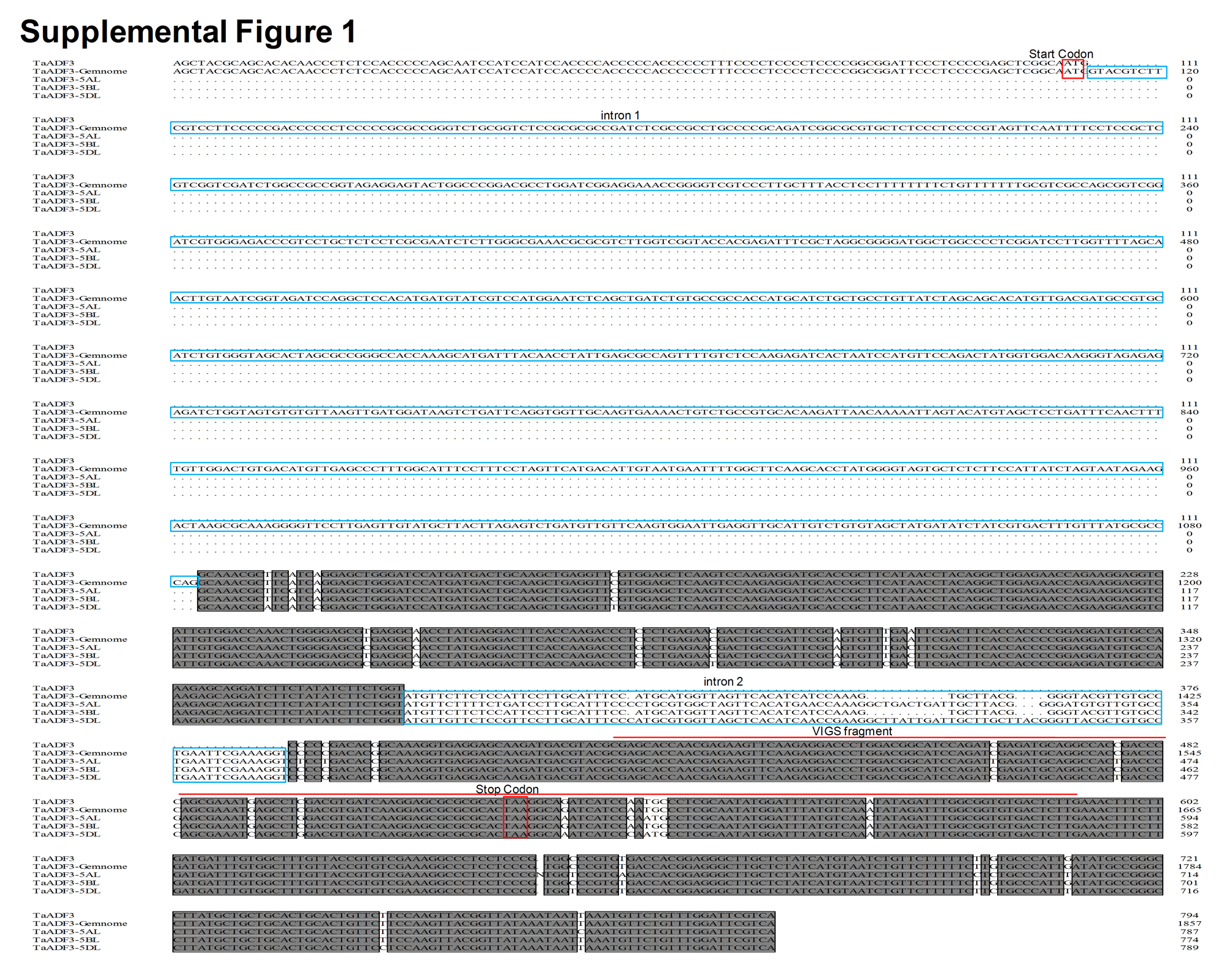

Supplement: Figure S1 — Gene structure of TaADF3. TaADF3, the full-length cDNA sequence amplified from cDNA of wheat Suwon 11; TaADF3-Genome, the genomic DNA sequence amplified from the total genomic DNA of wheat Suwon 11. The genome sequences of the three copies of TaADF3 on chromosomes 5AL, 5BL, and 5DL were obtained from the wheat UGRI genome database of the wheat cultivar Chinese Spring. The full genome sequence of TaADF3 contains three exons and two introns. The start codon and stop codon are indicated by red boxes, and the two introns are indicated by blue boxes. The specific fragment used for silencing is indicated by a red line. [file Image1.tif]

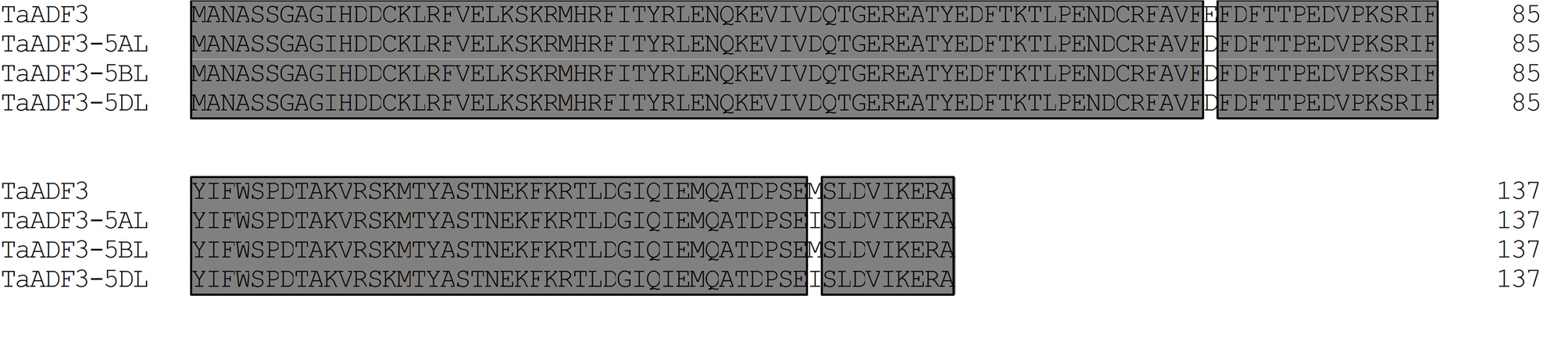

Supplement: Figure S2 — Multi-alignment of the deduced protein of the three copies of TaADF3. [file Image2.TIF]

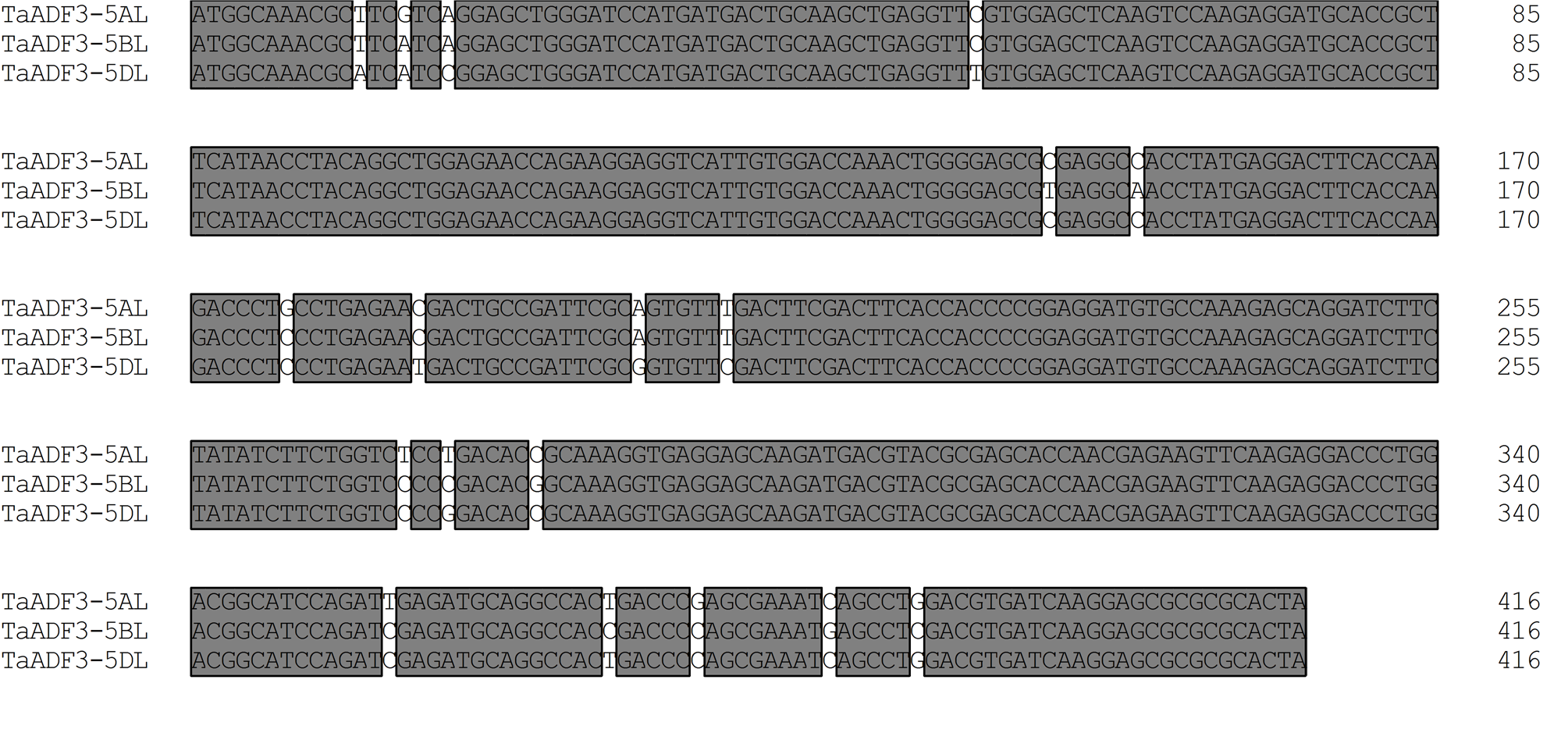

Supplement: Figure S3 — Multi-alignment of the encoding sequence of the three copies of TaADF3. [file Image3.TIF]

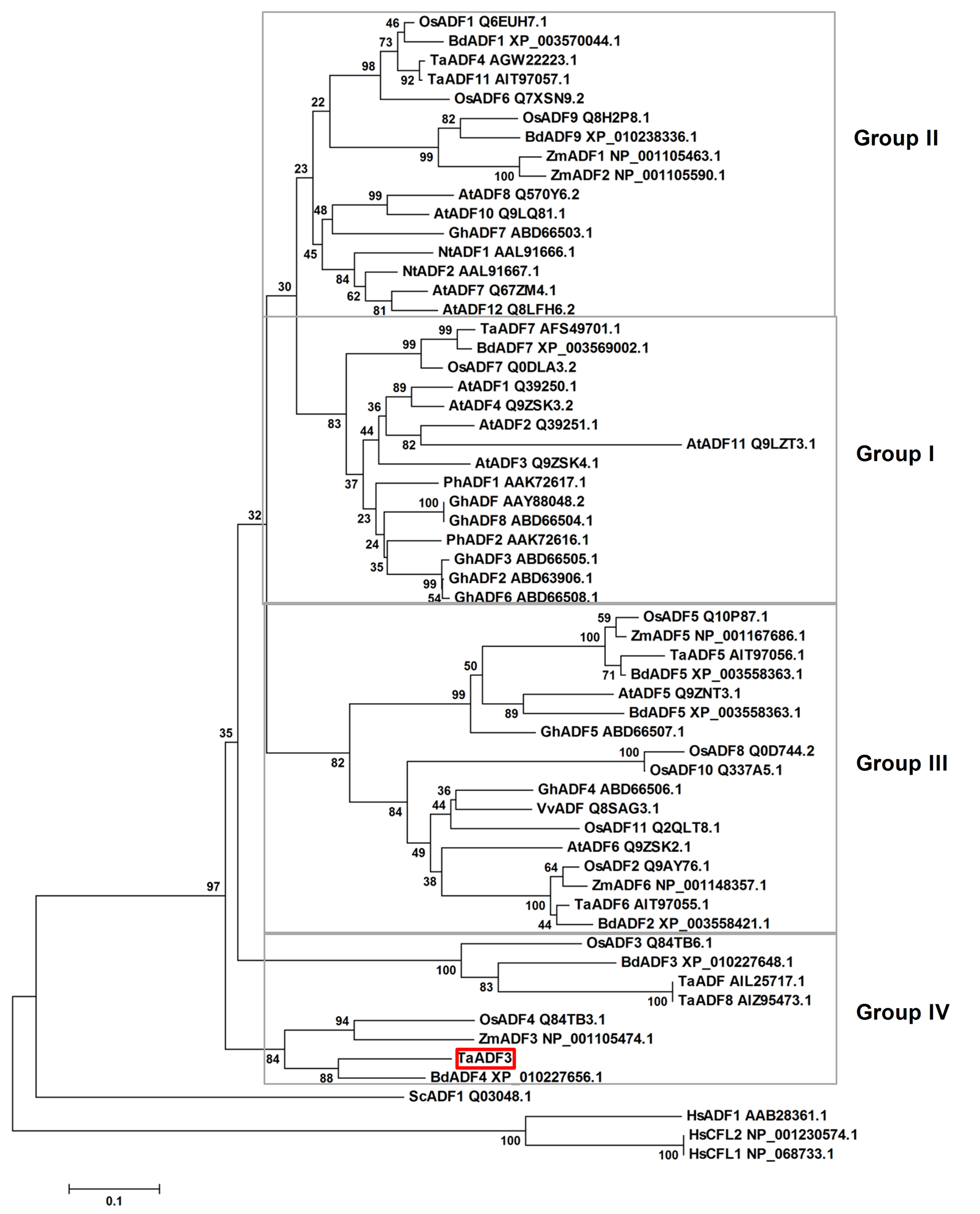

Supplement: Figure S4 — Phylogenetic analyses of TaADF3 and ADF members in other species. Branches are labeled with protein names and GenBank accession numbers. Ta, Triticum aestivum; Bd, Brachypodium distachyon; Os, Oryza sativa; Zm, Zea mays; At, Arabidopsis thaliana; Nt, Nicotiana tomentosiformis; Gh, Gossypium hirsutum; Ph, Petunia x hybrid; Sc, Saccharomyces cerevisiae; Hs, Homo sapiens. [file Image4.TIF]

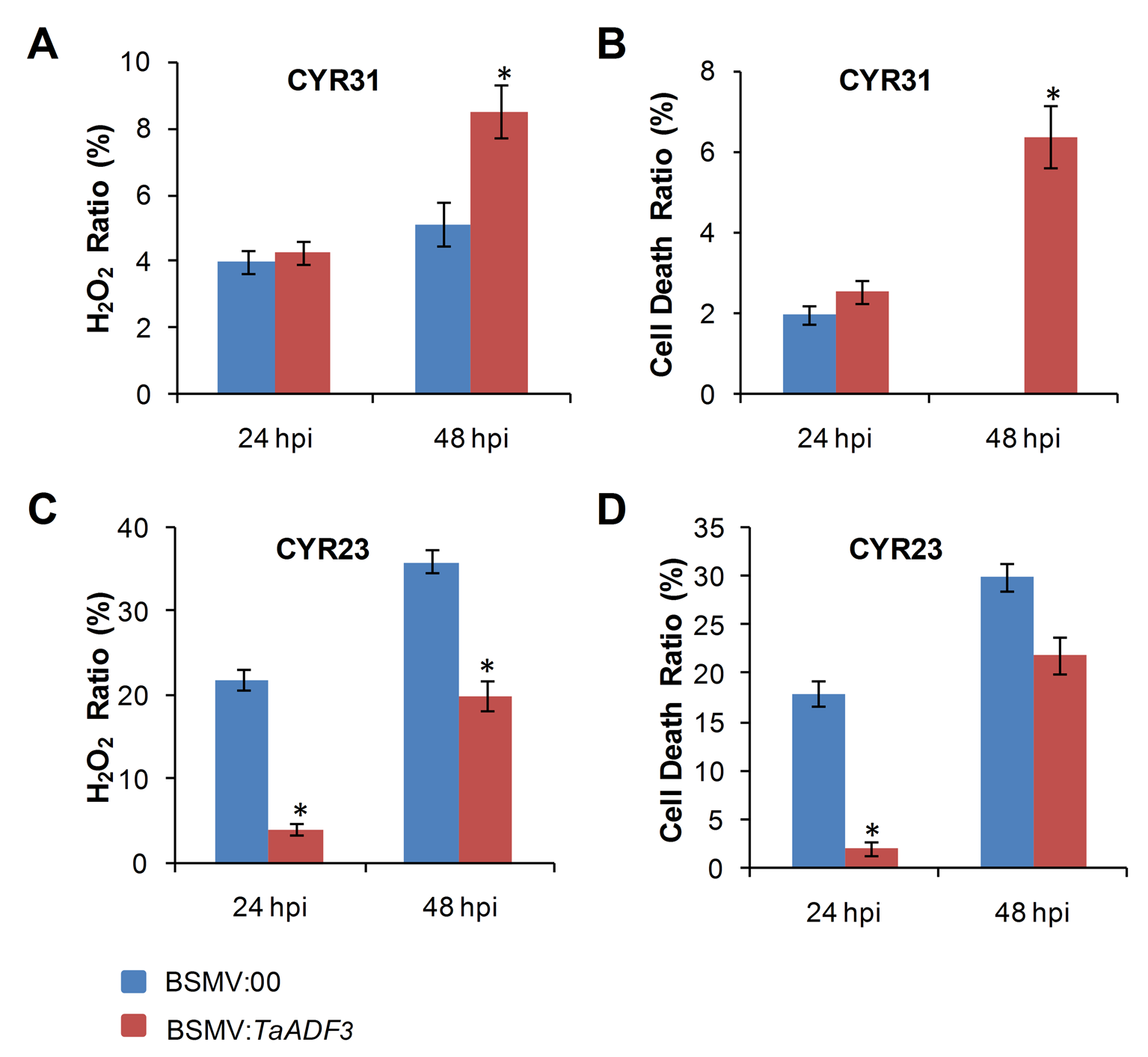

Supplement: Figure S5 — Incidence of H2O2 production and cell death in Pst infected TaADF3-knockdown plants. The fourth leaves of wheat seedlings inoculated with BSMV:00 or BSMV:TaADF3 were further challenged by virulent or avirulent Pst. The H2O2 accumulation in attacked mesophyll cells in each infection site was observed under differential interference contrast optics through DAB staining. Necrotic cell death of the mesophyll cells at the same infection site was viewed under a flurorescence microscope. The number of infection sites with H2O2 accumulation and necrosis in attacked mesophyll cells among 50 infection sites was calculated. The incidence of H2O2 production and necrosis in mesophyll cells attacked by virulent Pst CYR31 (A,B) or avirulent Pst CYR23 (C,D) was measured. Three independent biological replications were performed. Asterisks indicate a significant difference (P < 0.05) from BSMV:00 using Student's t-test. [file Image5.TIF]

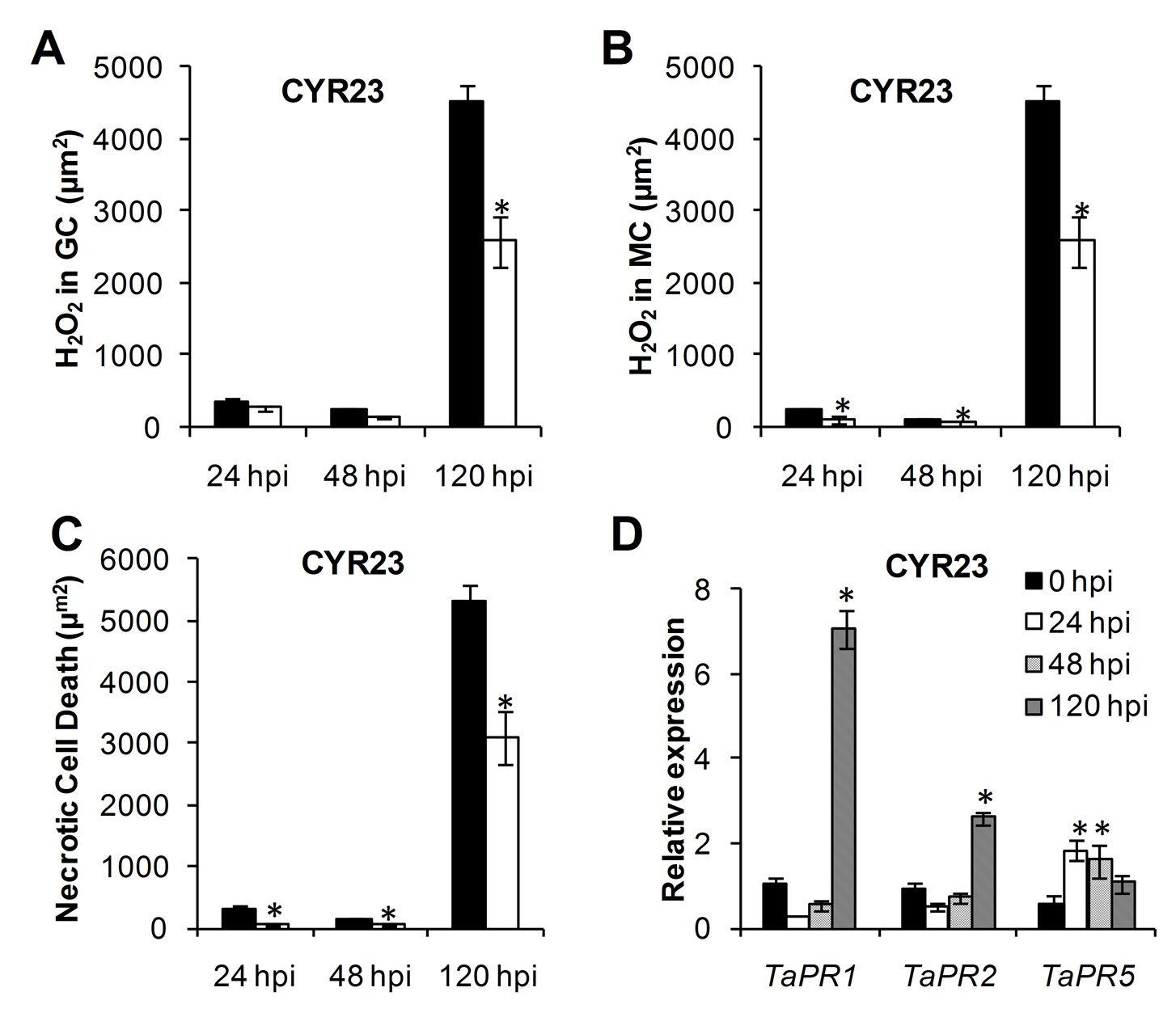

Supplement: Figure S6 — Decreased H2O2 production and cell death in TaADF3-knockdown plant challenged by avirulent Pst CYR23. The amount of H2O2 production was measured by calculating the DAB staining area at each infection site using DP-BSW software (A,B). The area of autofluorescence was measured to determine the necrotic cell death (C). H2O2 produced in guard cells (GC) and the attacked mesophyll cells (MC). All results were obtained from 50 infection sites. (D) The expression profiles of three pathogenesis-related proteins were assessed in TaADF3-knockdown plants compared withmock control plants. The data were normalized to the wheat TaEF-1α gene. Three independent biological replications were performed. Asterisks indicate a significant difference (P < 0.05) from BSMV:00 using Student's t-test. [file Image6.TIF]

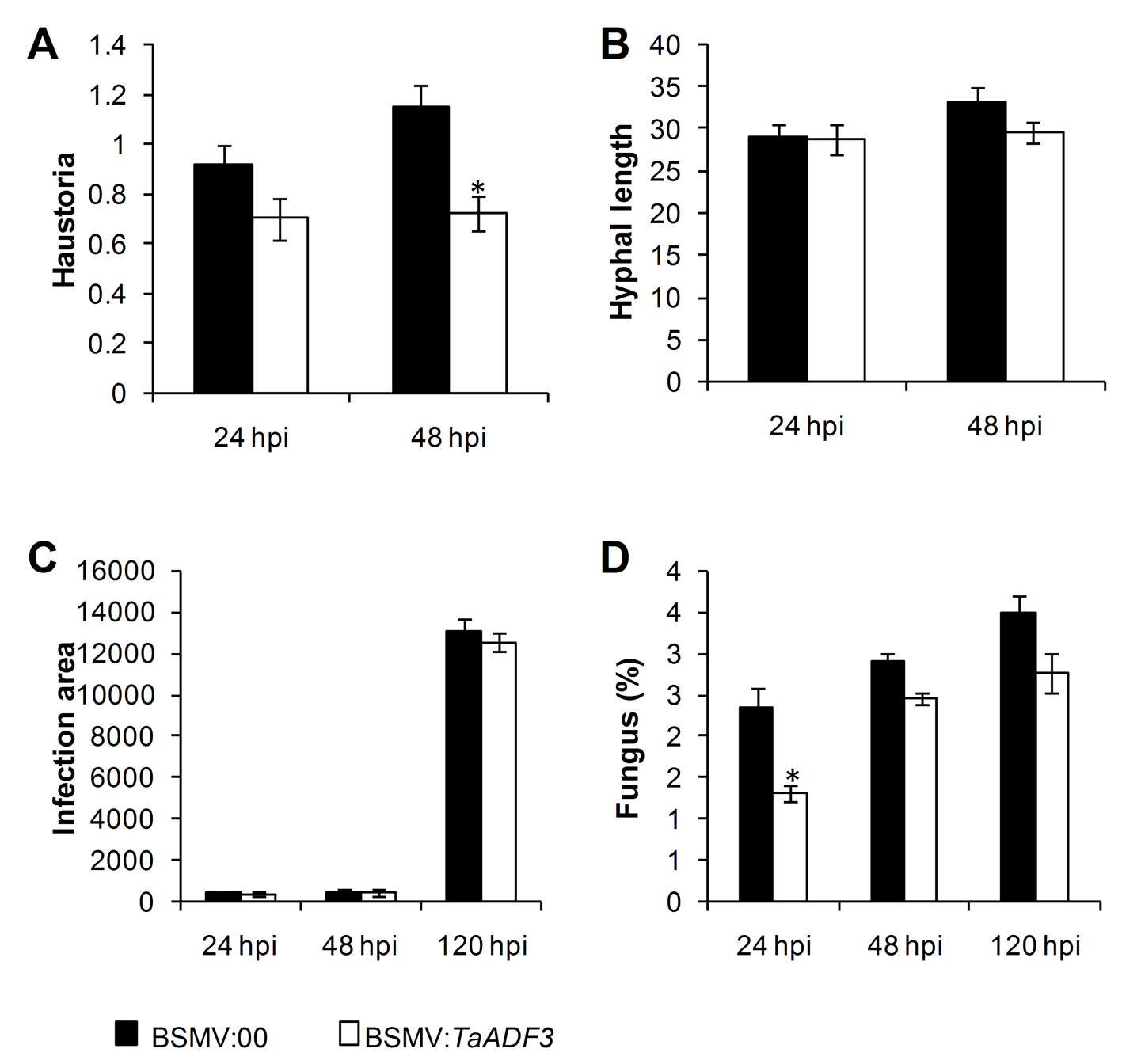

Supplement: Figure S7 — Fungal growth in TaADF3-knockdown plant challenged by avirulent Pst CYR23. (A) Average numbers of haustoria of Pst CYR23 in each infection site were counted. (B) Hyphal length, which is the average distance from the junction of the substomatal vesicle and the hypha to the tip of the hypha, was measured using DP-BSW software (unit in μm). (C) Infection area, the average area of the expanding hypha, was calculated using DP-BSW software (units of 103 μm2). All results were obtained from 50 infection sites, and three biological replications were performed. (D) Quantification of fungus in Pst-infected wheat leaves. The ratio of Pst CYR23 mRNA to total wheat mRNA was evaluated by qRT-PCR. Asterisks indicate a significant difference (P < 0.05) from BSMV:00-inoculated plants using a one-tailed Student's t-test. [file Image7.tif]
